# Aberrant Gcm1 expression mediates Wnt/β-catenin pathway activation in folate deficiency involved in neural tube defects

**DOI:** 10.1038/s41419-020-03313-z

**Published:** 2021-03-04

**Authors:** Jianting Li, Qiu Xie, Jun Gao, Fang Wang, Yihua Bao, Lihua Wu, Lihong Yang, Zhizhen Liu, Rui Guo, Ajab Khan, Dan Liu, Caihua Li, Jianxin Wu, Jun Xie

**Affiliations:** 1 Department of Biochemistry and Molecular Biology, Shanxi Medical University, 030001 Taiyuan, China; 2 Department of medical research center, Peking Union Medical College Hospital, Chinese Academy of Medical Sciences, 100730 Beijing, China; 3 Department of Postgraduate, Weifang Medical University, 261053 Weifang, China; 4 Beijing Municipal Key Laboratory of Child Development and Nutriomics, Capital Institute of Pediatrics, 100020 Beijing, China; 5 Department of Pathology, Shanxi Medical University, 030001 Taiyuan, Shanxi China; 6 Department of Bioinformatics, Genesky Biotechnologies Inc, 200120 Shanghai, China

**Keywords:** DNA, Nutrition disorders

## Abstract

Wnt signaling plays a major role in early neural development. An aberrant activation in Wnt/β-catenin pathway causes defective anteroposterior patterning, which results in neural tube closure defects (NTDs). Changes in folate metabolism may participate in early embryo fate determination. We have identified that folate deficiency activated Wnt/β-catenin pathway by upregulating a chorion-specific transcription factor Gcm1. Specifically, folate deficiency promoted formation of the Gcm1/β-catenin/T-cell factor (TCF4) complex formation to regulate the Wnt targeted gene transactivation through Wnt-responsive elements. Moreover, the transcription factor Nanog upregulated Gcm1 transcription in mESCs under folate deficiency. Lastly, in NTDs mouse models and low-folate NTDs human brain samples, *Gcm1* and Wnt/β-catenin targeted genes related to neural tube closure are specifically overexpressed. These results indicated that low-folate level promoted Wnt/β-catenin signaling via activating Gcm1, and thus leaded into aberrant vertebrate neural development.

## Introduction

Neural tube defects (NTDs) are severe birth defects thought to be associated with genetic and environmental factors^1^, resulted from the failure of neural tube closure during first trimester. Prepregnancy supplementation with folate can prevent 30–70% of NTDs^2^, but still the mechanism behind its prevention remains unclear.

The Wnt signaling pathway plays a prominent role in gastrulation and anteroposterior axis specification during embryonic development of the nervous system^3^. Wnt1 and Wnt3a are expressed at the dorsal midline of developing neural tube^4,5^. Unnormal levels of β-catenin in embryonic neural stem cells inhibit the proliferation of neural progenitor cells and promote their differentiation^6^. Aberrant canonical Wnt/β-catenin pathway signaling leads to defective anteroposterior patterning^7^. The transcriptional activity of genes targeted by Wnt signaling has been proven to be associated with neuronal proliferation and specification^6^. *GCM1* (glial cell missing 1), which encodes a chorion-specific transcription factor, has recently been identified as a novel target of β-catenin/TCF4 complex during regulation of the fusion of syncytiotrophoblast (ST) cells. The activation of Wnt signaling is essential for upregulation of Gcm1 and ST cell specification. Also, it has been reported that a feedback loop involving Gcm1 and Frizzled regulates trophoblast differentiation and chorionic branching morphogenesis^8^. Additionally, *Wnt10b* is involved in the activation of β-catenin/GCM1 pathway during the process of BeWo cell fusion after forskolin/hCG treatment^9^. These findings suggested that Gcm1 may be linked with Wnt signaling pathway, which can affect its activity to control cell fate.

*Gcm1* gene was first identified as determinant of the glial of *Drosophila*. In *Drosophila*, the onset of *gcm* expression functions is a binary switch in the developing nervous system and a master regulator of gliogenesis^10^. Further studies identified a DNA-binding domain (the gcm box) in the amino terminal region of *gcm* and a transactivation domain in the carboxy terminal portion^11^. The former is found in target genes that encode transcriptional activators of glial fate and transcriptional repressors of neural fate^12^. Sequence similarities between *Drosophila gcm1* and mammalian *Gcm1* are high in the gcm box^13^. Considering its importance in neurogenesis in *Drosophila* embryos, Gcm1 was thought to be indispensable for nervous system development in mammals. However, contrary to speculations, rare studies reported mammalian *Gcm* genes expression in the nervous system. Instead, Gcm1 is testified to be essential for human placental development^14^.

In this report, we showed that folate deficiency activates Wnt/β-catenin pathway by upregulating Gcm1 through formation of Gcm1/β-catenin/TCF4 complex. Moreover, the transcriptional activity of Wnt signaling is regulated through Wnt-responsive elements (WREs). We further showed that Gcm1 is strongly expressed in low-folate NTDs samples, which is accompanied by upregulation of Wnt/β-catenin targeted genes related to neural tube closure. Taking together, this study suggested a mechanism by which a signaling pathway can act dynamically to regulate Wnt gene transcriptional programs mediated by Gcm1 through folate metabolism in neurodevelopment of vertebrates.

## Results

### Folate deficiency activates Wnt/β-catenin signaling

**Fig. 1:**
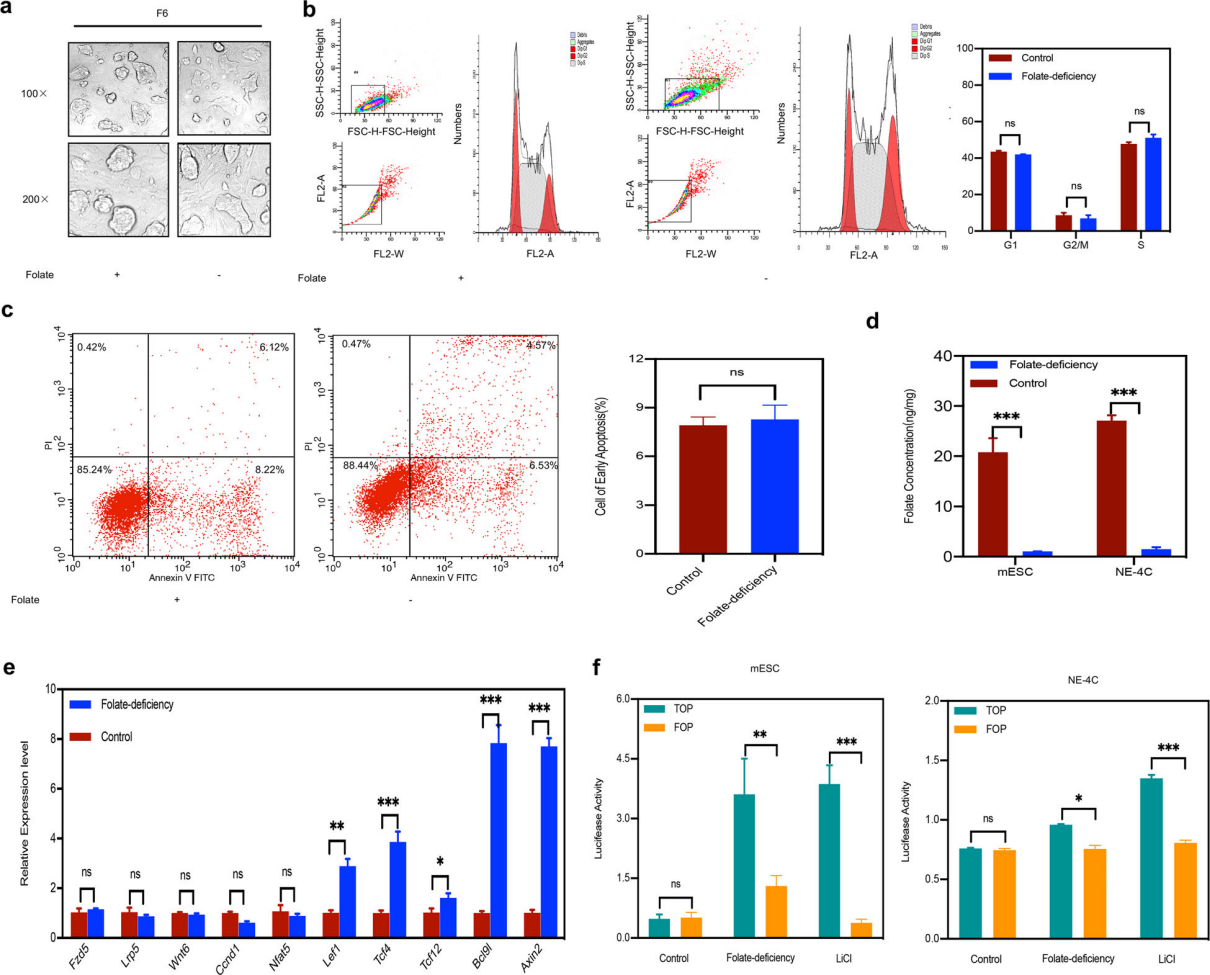
Wnt/β-catenin pathway signaling activation in cells with folate deficiency. **a** Morphology of C57BL/6 mESCs as observed with an optical microscope at magnifications of ×100 and ×200, F6, sixth generation of C57BL/6 mESCs. **b** Analysis of the cell cycle distribution of C57BL/6 mESCs after six generations of culture under folate deficiency by flow cytometry. 3 × 106 cells were harvested for control and case group respectively; right panel: the results of cell cycle quantification were graded according to the cell cycle phase. **c** Analysis of apoptosis in C57BL/6 mESCs after six generations of culture under folate deficiency by flow cytometry. 3 × 106 cells were harvested for control and case group, respectively, the number of cells in early apoptosis is shown in a bar graph in the right panel. **d** Folate concentration in C57BL/6 mESCs after six generations of culture in folate deficiency and in NE-4C after three generations of culture in folate deficiency. **e** The mRNA expression of *Fzd5, Lrp, Wnt6, Ccnd1, Nfat5, Lef1, TCF4, TCF12, Bcl9l*, and *Axin2* in C57BL/6 mESCs with folate deficiency. **f** Analysis of luciferase activity by TOP/FOP Flash assays in C57BL/6 mESCs subjected to folate deficiency for six generations and NE-4C subjected to folate deficiency for three generations. Data **a–f** represent the mean ± SEM (*n* = 3). The *p* value was calculated by Student’s *t*-test, ns was for no significance, *P < 0.05, **P < 0.01, ***P < 0.001.

Aberrant Wnt/β-catenin pathway signaling leads to defective anteroposterior patterning and thus results in NTDs^7^. To explore the potential effect of folate on Wnt/β-catenin pathway in NTDs, a folate-deficient C57BL/6 mESCs model was first established as previously reported^15^. Following sixth generation, no significant differences in the total numbers of cells or cell morphology were observed between the two groups (Fig. 1a). Comparison of cell cycle distribution and apoptosis indicated that arrival at the G_2_/M checkpoint was not delayed in the sixth generation of folate-deficient group (Fig. 1b), and no difference in apoptosis was observed (Fig. 1c). The critical indicator-folate concentration was with lower intracellular levels in folate-deficient mESCs (1.05 ± 0.03 ng/10^6^ cells) than in control mESCs (23.3 ± 1.78 ng/10^6^ cells). Additionally, in folate-deficient NE-4C cells for consecutive three generations, the folate concentration in control group was 27.06 ± 1.52 ng/10^6^ cells compared with folate-deficient group, which was 1.87 ± 0.19 ng/10^6^ cells (*P* < 0.01, Fig. 1d) with no obvious effect on cell survival. Next, we performed an Agilent Mouse mRNA Array with eight identical arrays per slide (8 × 60 K) on folate-deficient C57BL/6 mESCs, focusing on the expression of genes involved in the Wnt/β-catenin signaling pathway. Interestingly, real-time RT-PCR showed that β-catenin upstream genes in the Wnt signaling pathway exhibited no significant transcriptional changes in folate deficiency. However, the downstream target genes of β-catenin were significantly elevated (0.6–7-folds) (Fig. 1e). Meanwhile, the transcriptional activity of TOP/FOP was significantly activated under folate deficiency compared to the positive control group activated by LiCl (Fig. 1f). Altogether, our data presented the initial evidence that folate deficiency may activate the Wnt/β-catenin signaling pathway.

### Gcm1 regulates Wnt/β-catenin signaling activation by forming the Gcm1/β-catenin/TCF4 complex

**Fig. 2:**
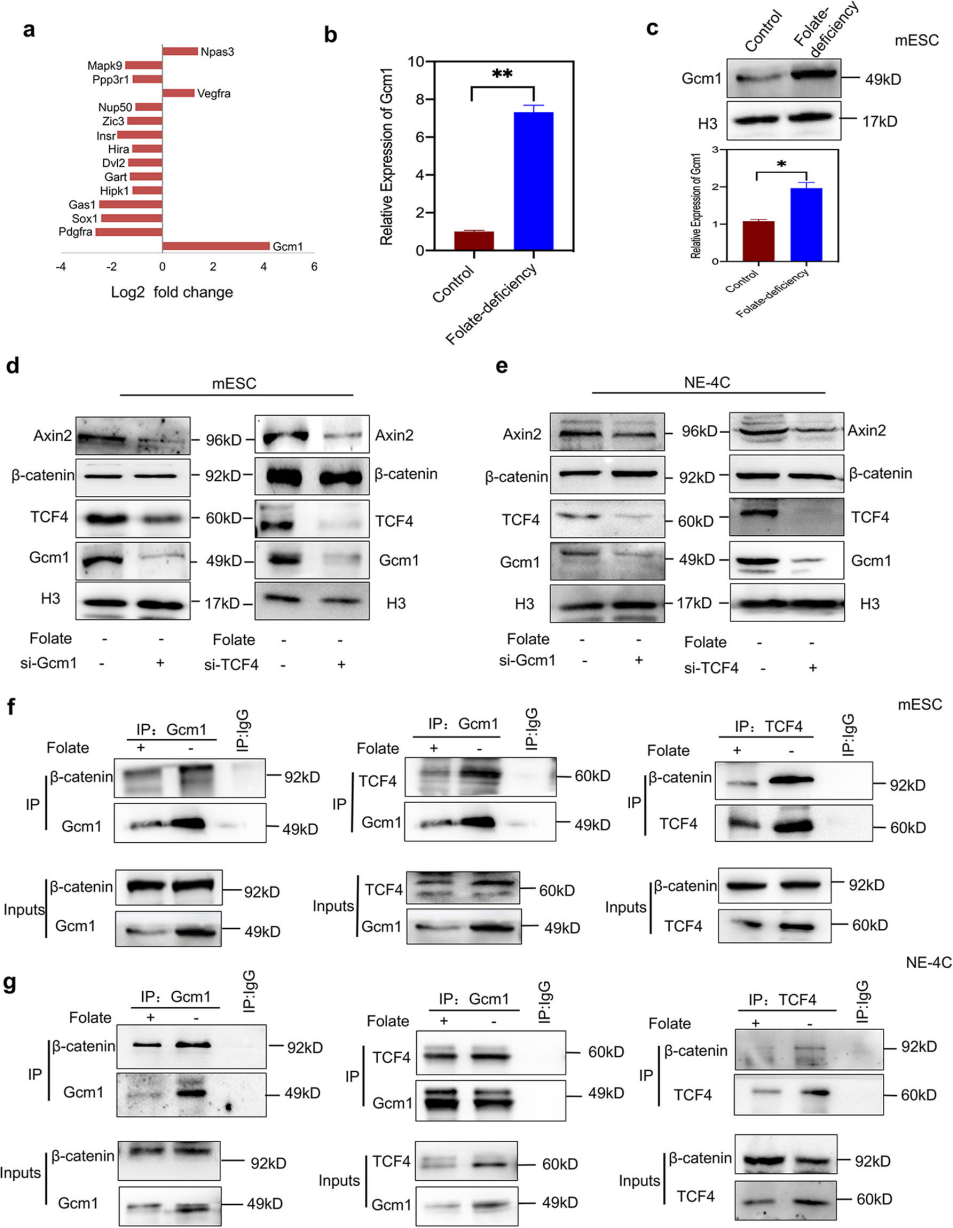
Folate deficiency upregulates Gcm1 to promote formation of the Gcm1/β-catenin/TCF4 complex. **a** Differentially expressed NTD-related genes in the microarray expression profiles of C57BL/6 mESCs cultured in folate-deficient conditions for six generations. The microarray data were obtained from three replicates for both control and folate-deficient cells. The *X*-axis represents the Log2 fold change. **b**The mRNA expression of *Gcm1* in C57BL/6 mESCs after six generations of folate deficiency. **P = 0.004. **c** Increased expression of Gcm1 in mESCs with folate deficiency. Top panel: western blots of Gcm1 in control and folate-deficient cells; bottom panel: bar graph showing the quantification of the western blot signal intensities. **d** Efficiency of siRNA-mediated Gcm1 and siRNA-mediated TCF4 depletion and the expression of TCF4, Gcm1 and Axin2 at the protein level in C57BL/6 mESCs. mESCs with folate deficiency were transfected into either scramble siRNA control or Gcm1 siRNA and TCF siRNA, and post 72 h of transfection, the expression was determined by western blotting. **e** Efficiency of siRNA-mediated Gcm1 and TCF4 depletion and the expression of TCF4 and Axin2 at the protein level in NE-4C. NE-4C with folate deficiency were transfected into either scramble siRNA control or Gcm1 siRNA, TCF4-siRNA, and the expression was determined 72 h post transfection by western blotting. **f** A coimmunoprecipitation (Co-IP) assay was used to identify the interaction between Gcm1, β-catenin, and TCF4 in C57BL/6 mESC. Immunoprecipitation (IP) was performed using an anti-Gcm1 antibody and TCF4 antibody, and immunoblotting (IB) was performed using an anti-β-catenin antibody. **g** A Co-IP assay was used to identify the interactions between Gcm1 and TCF4, β-catenin in NE-4C. An anti-Gcm1 antibody and anti-TCF4 antibody was used for IP, and an anti-TCF4 antibody and anti-β-catenin was used for IB. Data **b–f** represent the mean ± SEM (*n* = 3). The *p* value was calculated by Student’s *t*-test, ns was for no significance, *P < 0.05, **P < 0.01, ***P < 0.001.

To identify the potential mechanism of Wnt/β-catenin signaling activation with low-folate levels, we first analyzed NTD candidate genes (Table S1) expression through microarray expression profiling. Notably, *Gcm1* transcription was ranked the highest among all selected genes under folate deficiency (fold change > 2, *P* < 0.05, Table S2). The expression of *Gcm1* was upregulated 16-folds in folate-free conditions (Fig. 2a). Real-time RT-PCR and western blotting further confirmed that the relative *Gcm1* mRNA expression was significantly higher (*P* < 0.01, Fig. 2b) while the protein level was 2.5-folds higher (*P* < 0.05, Fig. 2c) compared with folate-deficient group. As shown in the database of the gene annotation portal BioGPS, the level of Gcm1 in normal mESCs is very low, which is consistent with our results (Fig. S1A). Considering that Gcm1 regulates cell fusion associated with Wnt/β-catenin signaling in syncytiotrophoblast cells^16,17^, we therefore investigated to know whether increased Gcm1 expression regulates Wnt/β-catenin in folate deficiency. Knockdown of Gcm1 showed that when 60% of the Gcm1 was inhibited (*P* = 0.03), the expression of β-catenin remained unchanged (*P* = 0.7), while the production of Axin2 and TCF4 were dramatically declined ~64% and 70%, respectively (for both *P* < 0.05, Fig. 2d, left panel). These results suggested that Gcm1 has affected the Wnt/β-catenin pathway activation, not by affecting the accumulation of β-catenin in nucleus but by preliminarily influencing TCF4 expression in the nucleus. For further confirmation, TCF4-siRNA parallel experiment was performed. The results corfirmed successful knockdown of TCF4, Gcm1, and Axin2 by 60%, 50%, and 55%, respectively, compared with the scramble control group (both *P* < 0.05, Fig. 2d, right panel). Similar results were also verified in folate-deficient NE-4C, with Gcm1 and TCF4 knockdown, which showed reduction in the expression of Axin2 while β-catenin was remained unchanged (Fig. 2e). The above data revealed that there was a feedback loop between Gcm1 and TCF4 expression level, which was independent of β-catenin accumulation in the nuclei with folate deficiency.

To specifically understand the regulation of Gcm1 in Wnt signaling activation with folate deficiency, coimmunoprecipitation (Co-IP) experiments were performed to explore the possible interactions among Gcm1, β-catenin and TCF4. Firstly, we found the level of β-catenin protein was unchanged in folate deficiency (*P* = 0.68, Fig. S1B). Remarkably, Co-IP assay showed a weak and strong Gcm1/β-catenin binding in the control and folate-deficient groups, respectively, (Fig. 2f, left panel). Since it has been confirmed that TCF4 plays a pivotal role in the activation of Wnt/β-catenin pathway^18,19^, we also performed Co-IP assay between Gcm1 and TCF4 in folate deficiency. The results showed an increase in binding between Gcm1 and TCF4 in folate deficiency (Fig. 2f, middle panel). To further validate whether there is also binding between β-catenin and TCF4 in folate deficiency, a Co-IP assay with antibody against TCF4 was performed. The results confirmed a detectable increased in the binding between β-catenin and TCF4 in folate deficiency (Fig. 2f, right panel). Similarly, same Co-IP assays in NE-4C revealed an increase in Gcm1 combining with β-catenin and TCF4, respectively, in folate-deficient condition (Fig. 2g). Taken together, these results suggested that folate deficiency had significantly regulated the formation of Gcm1, TCF4, and β-catenin tri-complex.

**Fig. 3:**
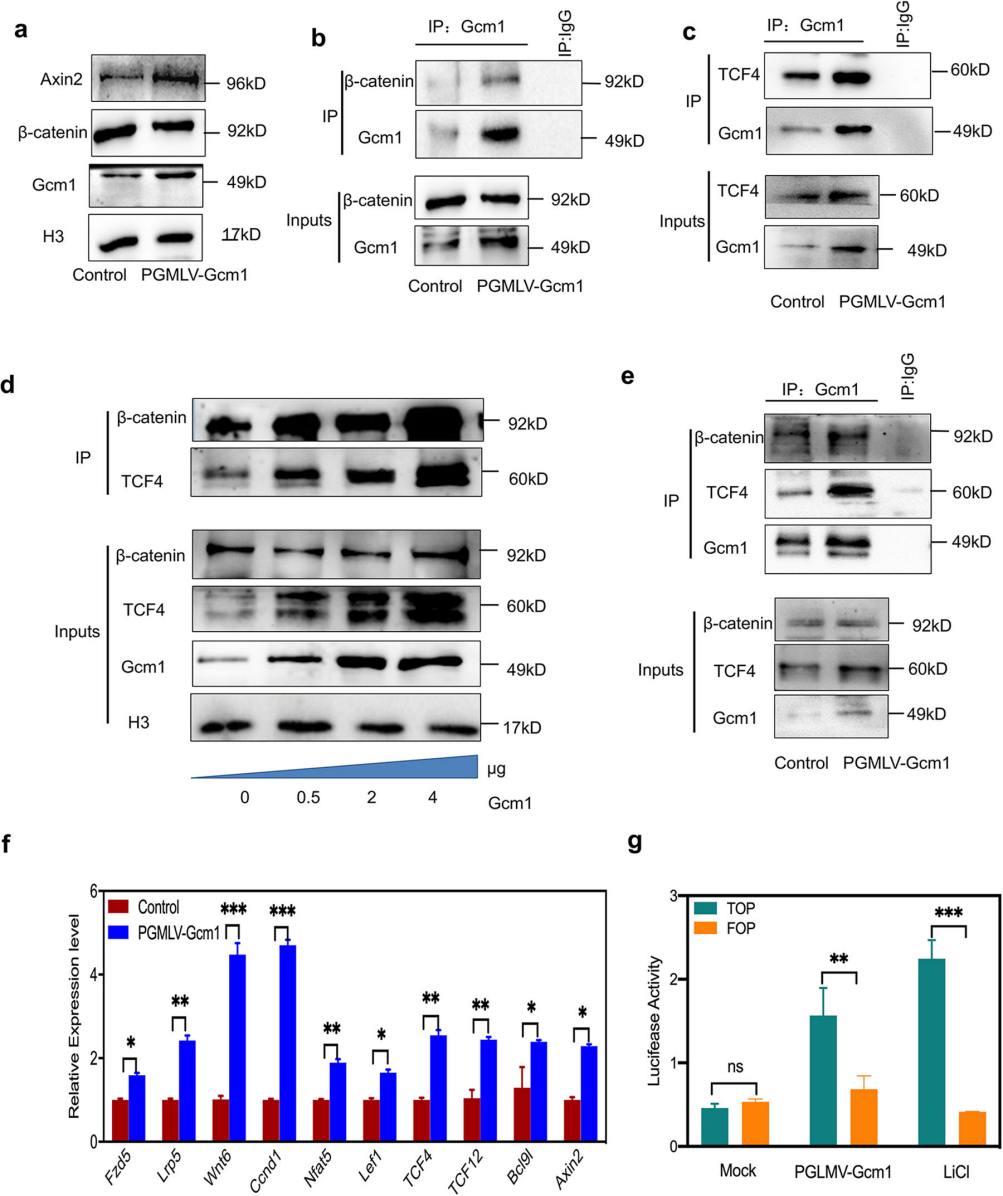
Gcm1 overexpression activates the Wnt/β-catenin pathway in normal C57BL/6 mESCs and NE-4C. **a** Increased expression of Axin2 in mESCs with Gcm1 overexpression. The western blots show that the Gcm1 overexpression plasmid increased Axin2 expression in normal C57BL/6 mESCs. PGMLV-Gcm1, Gcm1 plasmid. mESCs were transfected with either control siRNA or pGMLV-Gcm1, and the expression levels were determined 72 h post transfection by western blotting. **b** A Co-IP assay was used to identify the interaction between Gcm1 and β-catenin in cells transfected with pGMLV-Gcm1 overexpression plasmid in C57BL/6 mESCs. An anti-Gcm1 antibody was used for IP, and IB was performed using an anti-β-catenin antibody. **c** A Co-IP assay was used to identify the interaction between Gcm1 and TCF4 in cells transfected with pGMLV-Gcm1 overexpression plasmid in C57BL/6 mESCs. An anti-Gcm1 antibody was used for IP, and IB was performed using an anti-TCF4 antibody. **d** A Co-IP assay identified increase interactions between TCF4 and β-catenin in cells transfected with 0.5, 2, and 4 µg of pGMLV-Gcm1 for 72 h in C57BL/6 mESCs. **e** A Co-IP assay was used to identify the interaction between Gcm1 and TCF4, β-catenin in NE-4C cells transfected with the pGMLV-Gcm1 overexpression plasmid. An anti-Gcm1 antibody was used for IP, and IB was performed using an anti-TCF4 antibody and anti-β-catenin antibody. **f** The mRNA expression of *Fzd5, Lrp, Wnt6, Ccnd1, Nfat5, Lef1, Tcf4, Tcf12, Bcl9L*, and *Axin2* in C57BL/6 mESCs transfected with the overexpression vector pGMLV-Gcm1. The mRNA was extracted from the mESCs after transfection with pGMLV-Gcm1 for 72 h. **g** Analysis of luciferase activity by TOP/FOP Flash assays in C57BL/6 mESCs transfected with pGMLV-Gcm1 overexpression plasmid relative to those transfected with scramble plasmid in normal and LiCl medium. LiCl (10 mmol) was added to the control medium before 72 h of transfection. Data **a–g** represent the mean ± SEM (*n* = 3). The *p* value was calculated by Student’s *t*-test, ns was for no significance, *P < 0.05, **P < 0.01, ***P < 0.001.

Based on our observation, folate deficiency induced an increased in endogenous Gcm1 expression, which further activated Wnt signaling. Furthermore, we also investigated whether exogenous overexpression of Gcm1 would have a similar effect. Post 72 h of Gcm1 plasmid (PGMLV-Gcm1) transfection, both Gcm1 and Axin2 expressions were significantly increased, while β-catenin expression was remained unchanged (*P* = 0.53, Fig. 3a). The Co-IP assay using an antibody against Gcm1 showed an overexpression of Gcm1 which promoted self-binding with β-catenin and TCF4 (Fig. 3b and c). Additionally, the interaction between between β-catenin and TCF4 was significantly increased in the presence of PGMLV-Gcm1 in a dose-dependent manner (Fig. 3d). Lastly, similar strong binding between Gcm1, TCF4, Gcm1, and β-catenin were also verified in the NE-4C with Gcm1 overexpression (Fig. 3e). Collectively, all the data demonstrated that overexpression of Gcm1 promoted Gcm1/β-catenin/TCF4 complex formation.

It is well known that β-catenin binds to TCF4, resulting in the activation of Wnt target gene upon Wnt stimulation^19^.Thus we hypothesized that Gcm1/β-catenin/TCF4 complex may also play a role in the activation of Wnt signaling when Gcm1 is overexpressed. Real-time RT-PCR demonstrated that the expressions of relative genes (shown in Fig. 1e) were significantly increased (2-folds) (Fig. 3f). The transcription of TOP/FOP exhibited 2.4-fold higher level with Gcm1 overexpression, which was consistent with the positive control group treated with LiCl (Fig. 3g). Together, these results suggested that an aberrant increased in Gcm1 expression activated Wnt signaling by mediating Gcm1/β-catenin/TCF4 tri-complex formation.

### Gcm1 modulates Wnt target genes coordination with β-catenin/TCF4 in folate deficiency

**Fig. 4:**
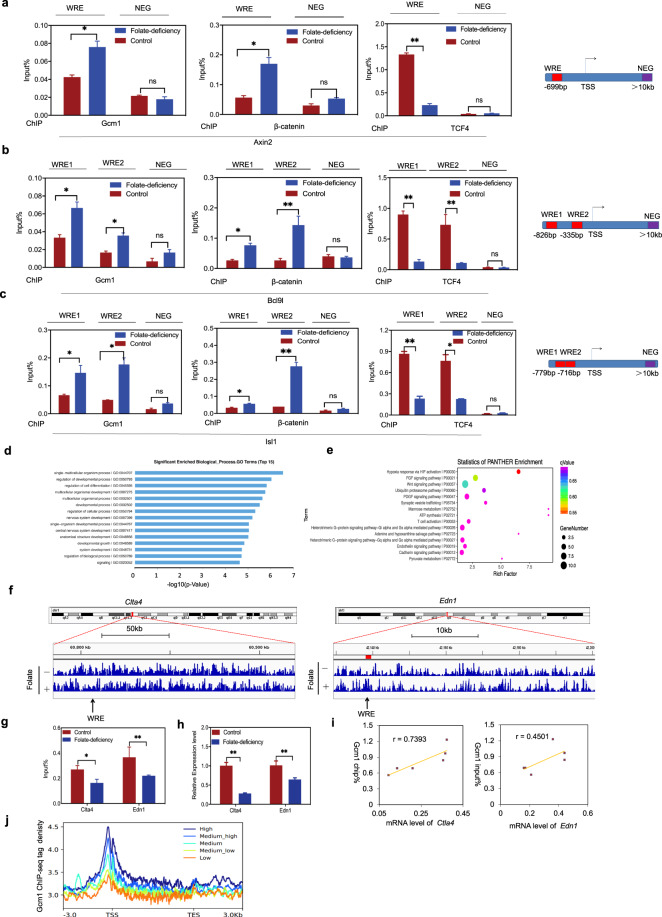
Gcm1 competes with β-catenin for binding to TCF4 and is associated with Wnt target genes expression. **a** ChIP-qPCR analysis of Gcm1, β-catenin and TCF4 enrichment in Wnt-responsive element (WREs) of *Axin2* in control and folate-deficient mESCs. NEG negative control, TSS transcription start site. Right panel: the locations of the WRE and NEG in the promoter. **b** ChIP-qPCR analysis of Gcm1, β-catenin, and TCF4 enrichment in the WREs of *Bcl9l* in control and folate-deficient mESCs. Right panel: the locations of the WREs and NEG in the promoter. **c** ChIP-qPCR analysis of Gcm1, β-catenin, and TCF4 enrichment in the WREs of *Isl1* in control and folate-deficient mESCs. Right panel: the locations of the WREs and NEG in the promoter. **d** Functional clustering of genes associated with Gcm1 ChIP-peaks in mESCs with folate deficiency (the top 10 categories are shown). The *X*-axis represents the log10 value of the binomial raw *P* value. **e** Pathway analysis of Gcm1 ChIP-peaks in mESCs with folate deficiency (the top 10 categories are shown). Gcm1 binding pathway enrichment was determined using PANTHER analysis. The *X*-axis represents the enrichment factor, the *Y*-axis represents the binomial raw *P* value, and the size of each circle represents the number of genes. **f** Representative examples of *Ctla4* and *Edn1* chromatin binding. A segment of chromosome 1 demonstrates the decreased binding of *Clta4* to a TBE in folate-deficient compared to normal mESCs. A segment of chromosome 13 demonstrates the decreased binding of *Edn1* to a TBE in folate-deficient group compared to normal mESCs. **g** ChIP-qPCR analysis of *Clta4* and *Edn1* in mESCs with folate deficiency. *P = 0.015 (*Clta4*) and *P = 0.022 (*Edn1*). **h** The mRNA expression of *Clta4* (J) and *Edn1* (K) in mESCs with folate deficiency. *P = 0.015 (*Clta4*) and *P = 0.04 (*Edn1*). **i** Correlation analysis between ChIP density and the mRNA expression of *Clta4* and *Edn1*. The correlation coefficients for *Clta4* is 0.739 (*P* = 0.003), and for *Edn1* is 0.45 (*P* = 0.002). **j** Correlation of the tag densities (*Y*-axis) with the transcription levels of genes from −3 kb to +3 kb (*X*-axis). All the genes were divided into five groups based on their transcription levels from high (dark blue) to low (orange). The mean-normalized ChIP-seq densities with 1-bp resolution are plotted within a 6-kb region flanking the TSS or the TTS (transcription termination site). Data **a–c**, **g–i** represent the mean ± SEM (*n* = 3). The *p* value was calculated by Student’s *t-*test, ns was for no significance, *P < 0.05, **P < 0.01, ***P < 0.001.

The β-catenin/TCF4 complex activates Wnt signaling by binding to WREs (5′-CAAAGG-3′) in the promoter regions of Wnt target genes to activate their expression^19^. Keeping this in view, we examined whether Gcm1/β-catenin/TCF4 tri-complex could bind to the promoter WREs of Wnt target genes to control gene expression patterns. ChIP-qPCR experiments with antibodies against Gcm1, β-catenin and TCF4 were performed to assess Wnt targeted genes (*Axin2*, *Bcl9l*, and *Isl1*). A region located 10-kb downstream of the transcription start site (TSS) of the Wnt target gene was examined as a negative control (NEG). As shown in Fig. 4a, ChIP-qPCR demonstrated that folate deficiency enhanced (2-folds) Gcm1 enrichment and (3-folds) β-catenin enrichment at the WRE sequence of *Axin2* in folate-deficient group (*P* < 0.05). Unexpectedly, the enrichment of TCF4 at the WRE of *Axin2* was reduced to nearly 5-fold in folate-deficient group (*P* < 0.01). These results suggested that the binding pattern of the Gcm1/β-catenin/TCF4 tri-complex in *Axin2* changed depending on the folate level. Similar findings were observed for *Bcl9l* and *Isl1* with folate deficiency (Fig. 4b and c). In summary, all these findings revealed that Gcm1/β-catenin/TCF4 tri-complex specifically bound to WREs of Wnt target genes and orchestrated Wnt target gene activation in folate-deficient conditions.

To explore whether Gcm1 was involved in modulating the transcription of Wnt target genes in folate deficiency, we performed genome-wide ChIP-seq of Gcm1 in mESCs to determine its binding characteristics. Gene Ontology (GO) analysis showed that the top biological processes overlapped and included developmental processes and nervous system development (Fig. 4d). Notably, PANTHER pathway analysis showed a remarkable number of genes associated with Gcm1 enrichment were also associated with the Wnt signaling pathway (Fig. 4e). Next, we investigated that how enrichment of Gcm1 overlapped with Wnt target genes and affect their expression. From ChIP-seq, in the Gcm1 peaks, two Wnt target genes (*Ctla4* and *Edn1*), which are considered as necessary for the neural tube closure process, were selected as representatives (Fig. 4f). The WRE sequence of *Ctla4* and *Edn1* both exhibited decrease in intensity at the Gcm1 peak in folate deficiency. ChIP-qPCR also verified that Gcm1 interaction at *Ctla4* WREs and *Edn1* WREs were decreased to approximately 65% and 61%, respectively (*P* < 0.01, Fig. 4g). Furthermore, the mRNA expression of *Ctla4* and Edn1 were also (72% and 64%, respectively) lowered in the folate-deficient group (*P* < 0.01, Fig. 4h). Correlation analysis showed a significant positive correlation between Gcm1 enrichment and *Ctla4* and *Edn1* mRNA expression (Fig. 4i). These results implied that Gcm1 enrichment was associated with the transcription of Wnt target genes in folate deficiency. Next, the overall peaks of Gcm1 were further combined with the microarray data of both control and folate-deficient mESCs. The results conclusively demonstrated that there was a positive correlation between Gcm1 enrichment and gene expression. Higher Gcm1 enrichment near the TSS region was associated with higher gene expression (Fig. 4j). Overall these data indicated that the Gcm1/β-catenin/TCF4 tri-complex bound to Wnt target genes in folate deficiency, in which Gcm1 had specifically linked to Wnt target promoters.

### Nanog upregulates Gcm1 transcription with folate deficiency

To understand how folate level affect Gcm1 expression, we performed the DNA methylation of *Gcm1* with folate deficiency, considering that folate serves as a source of epigenetic modifications in S-adenosylmethionine (SAM)-mediated one-carbon transfer reactions. The results showed that there were no variations in five CpG sites in the *Gcm1* promoter region (Fig. S3A). ChIP-qPCR experiments using antibodies against H3K27me3 and H3K4me3 also showed no differences between the two groups as shown in Fig. S3B.

**Fig. 5:**
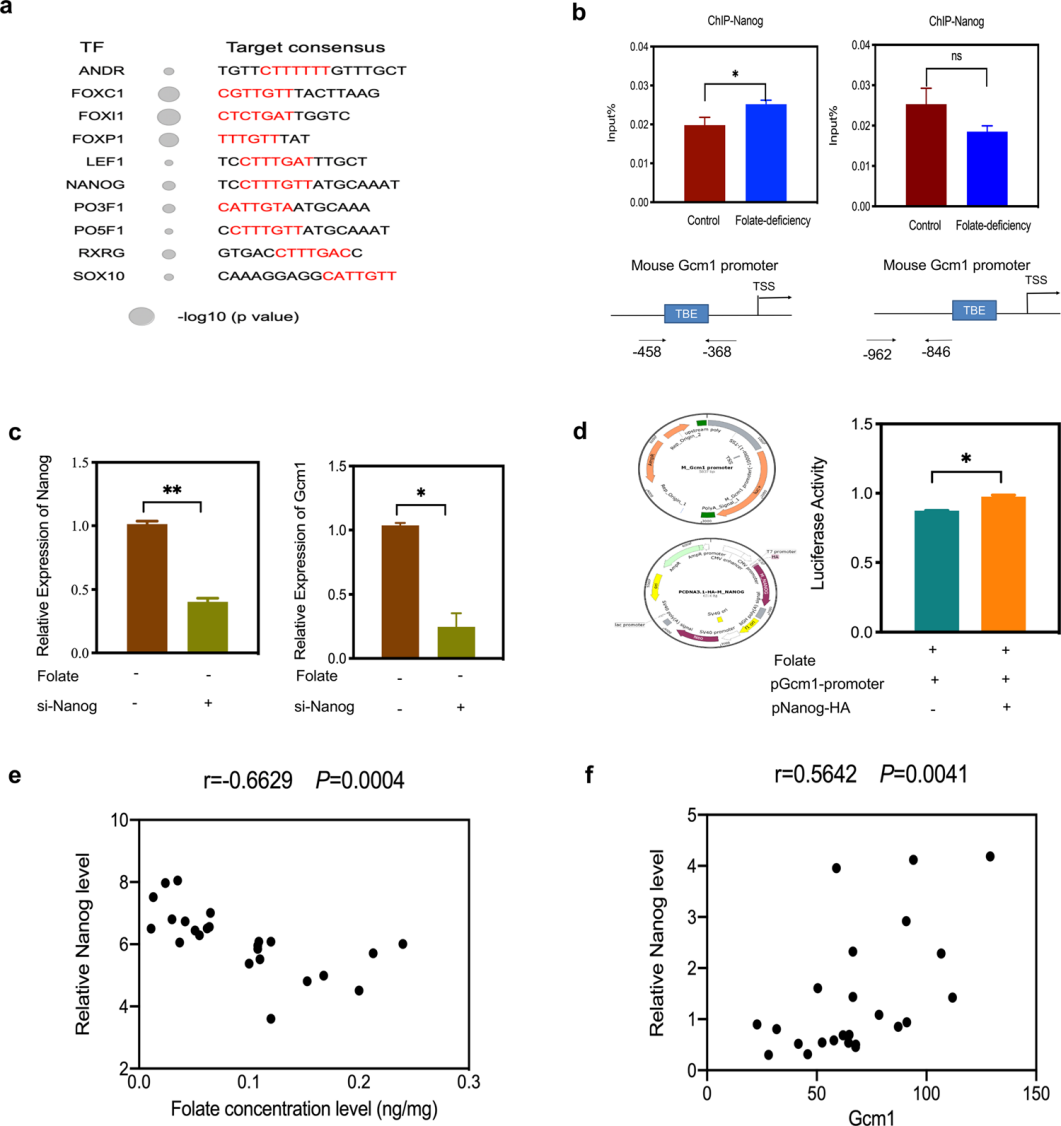
Nanog upregulates Gcm1 promoter activity. **a** Enrichment scores of transcription factor binding motifs in the mouse Gcm1 promoter. TF transcription factor. The size of the circle represents the −log10 (*P* value). **b** Nanog recognizes the *Gcm1* promoter. C57BL/6 mESCs were subjected to ChIP analysis using Nanog antibodies. The immunoprecipitated complexes were analyzed by PCR. The TBE sites are located within 1 kb upstream of the *Gcm1* promoter; the arrows represent the start and end sites of the PCR amplicons (bottom panel). **c** Efficiency of siRNA-mediated Nanog depletion and the expression of *Gcm1* at mRNA level. C57BL/6 mESCs with folate deficiency were transfected with either control scramble siRNA or Nanog-siRNA, and the expression levels were determined 24 h post transfection by real-time RT-PCR. Left panel: quantification of Nanog in control scramble siRNA and si-Nanog cells; right panel: quantification of Gcm1 in control scrambles siRNA and si-Nanog cells. **d** Analysis of Gcm1 promoter-driven luciferase reporter activity in folate-deficient mESCs cotransfected with control plasmid or Nanog plasmid. Experimental mESCs were transfected with 1.5 µg of pGcm1-promoter, and control mESCs were transfected with 5.5 µg of Nanog-HA. The structures of the Gcm1 promoter reporter construct and the Nanog-HA construct are shown in the left panel. At 48 h post transfection, the cells were harvested for luciferase reporter assays. **e** Correlation analysis of folate concentration and *Nanog* mRNA expression in low-folate NTDs (*n* = 12). **f** Correlation analysis of Nanog mRNA expression and *Gcm1* mRNA expression in low-folate NTDs, determined by Nanostring (*n* = 12). Data **b–d** represent the mean ± SEM (*n* = 3). Data **e, f** represent the mean mean ± SEM (*n* = 12). The *p* value was calculated by Student’s *t*-test, ns was for no significance, *P < 0.05, ** *P* < 0.01, ***P < 0.001.

To further investigate the underlying mechanism of upregulated Gcm1 transcription in folate deficiency, candidate transcription factor binding motifs near the *Gcm1* promoter were screened. As Nanog binding motifs harboring TBEs are enriched within a 1 kilobase (kb) region of the *Gcm1* promoter, the impact of Nanog on *Gcm1* gene expression in folate deficiency was investigated (Fig. 5a). Firstly, ChIP-qPCR showed folate deficiency promoted Nanog binding in a region harboring TBE motifs in the promoter of *Gcm1* (*P* < 0.05, Fig. 5b). Next siRNA-Nanog experiment indicated knockdown of *Nanog* reduced *Gcm1* expression at the mRNA level (*P* < 0.05, Fig. 5c). For further confirmation, we tested whether Nanog upregulates *Gcm1* promoter activity in mESCs, a luciferase assay was performed. As illustrated in Fig. 5d, after co-expression with pGcm1-promoter, Nanog-HA consistently and significantly stimulated the luciferase activity driven by pGcm1-promoter, suggesting that Nanog was required for Gcm1 promoter activity. In addition, a correlation analysis of folate concentration and *Nanog* mRNA expression in human NTDs samples also showed a significant negative correlation between them (*P* < 0.001, Fig. 5e). However, there was a significant positive correlation between *Nanog* mRNA expression and *Gcm1* mRNA expressions in low-folate NTDs samples, which indicated that Nanog activated the transcription of *Gcm1* in low-folate NTDs samples (*P* < 0.001, Fig. 5f). Overall, these results demonstrated that Nanog was a critical player in the control of Gcm1 expression with folate deficiency.

### Gcm1 and Wnt target genes upregulation in NTDs

**Fig. 6:**
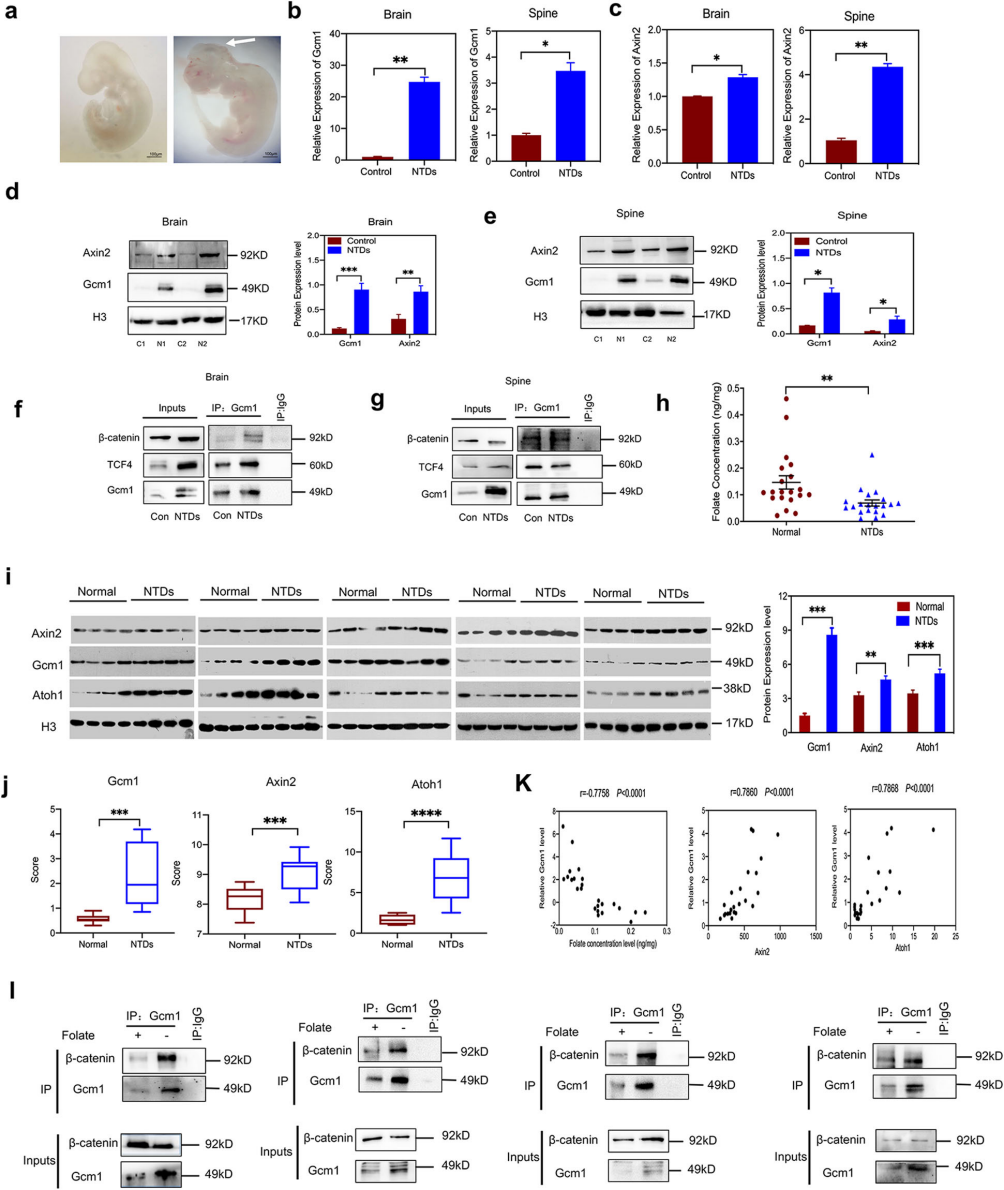
Gcm1 and Axin2 are significantly upregulated in NTDs models. **a** Morphology of CD-1 mouse embryos with low-folate diet and MTX induced from E9.5, left panel is control mouse fetus, right panels are NTDs mouse fetuses. The arrowhead is where the NTDs located; the left panel of NTDs embryos is schizencephaly. Scale bars, 100 μm. **b** The mRNA expression of *Gcm1* in the brains and spines of NTD model mice. *P = 0.0003(brains) and *P = 0.01 (spines). **c** The mRNA expression of *Axin2* in the brains and spines of NTD model mice. *P = 0.002(brains) and **P = 0.02 (spines). **d** Increased expression of Gcm1 and Axin2 in NTD mouse brains. Left panel: western blots of Gcm1 in normal and NTD brains; right panel: bar graph showing the quantification of the western blot signal intensities. **e** Increased expression of Gcm1 and Axin2 in NTD mouse spines. Left panel: western blots of Gcm1, Axin2 in normal and NTD brains; right panel: bar graph showing the quantification of the western blot signal intensities. **f** A Co-IP assay was used to identify the interaction between Gcm1 and TCF4, β-catenin in control and NTDs mouse brains. An anti-Gcm1 antibody was used for IP, and an anti-TCF4 antibody and anti-β-catenin was used for IB, Con was for Control. **g** A Co-IP assay was used to identify the interaction between Gcm1 and TCF4, β-catenin in control and NTDs mouse spines. An anti-Gcm1 antibody was used for IP, and an anti-TCF4 antibody and anti-β-catenin was used for IB, Con was for Control. **h** Folate concentrations in control human fetal brain tissue and spina bifida human fetal brain tissue. The data were obtained from 40 human fetus brains. **i** Increased expression of Gcm1, Axin2, and Atoh1 in human NTD brain samples. Left panel: western blots of Gcm1 in brains of normal humans (*n* = 20) and human NTD (*n* = 20) samples; right panel: bar graph showing the quantification of the western blot signal intensities. **j** The mRNA expression of *Gcm1*, *Axin2*, and *Atoh1* in the NTDs fetuses, determined by Nanostring (*n* = 12). **k** Correlation analysis of folate concentration and Gcm1 mRNA expression (left panel); *Gcm1* mRNA expression and *Axin2 mRNA expression*, *Atoh1* mRNA expression (right panel) *n* = 12. **l** A Co-IP assay identified an interaction between Gcm1 and β-catenin in randomly selected low-folate fetal brains. Data **a–h**, **l** represent the mean ± SEM (*n* = 3). Data **i** represent the mean ± SEM (*n* = 20), Data **j, k** represent the mean ± SEM (*n* = 12), The *p* value was calculated by Student’s *t*-test, ns was for no significance, *P < 0.05, **P < 0.01, ***P < 0.001.

Now that it was determined that Gcm1 was overexpressed to hyperactivate Wnt/β-catenin signaling, we aimed to determine whether the expression of *Gcm1* and *Axin2* were affected in NTDs mouse models. The NTDs mouse models used was the same as described previously in our laboratory^20,21^. Mouse nervous system development starts on day 7.5 and get completed on day 10.5 of embryonic development^22,23^ Keeping this in view, neural tissues were examined on day E9.5, because at this time point the nervous development is still in progress. The intact morphology of normal mouse embryos and the morphology of NTDs mouse embryos on day E9.5 were shown in Fig. 6a. Real-time RT-PCR showed that the expression of *Gcm1* was significantly increased in the brains and spines of NTDs mouse embryos (both *P* < 0.01, Fig. 6b). Similarly, *Axin2* in the brains and spines of NTDs mouse embryos were also increased at mRNA level (both *P* < 0.05, Fig. 6c). Notably, the expression of Gcm1 and Axin2 proteins level in the brains and spines of NTDs mouse embryos were much higher than that in normal tissue (Fig. 6d and e). Finally, the Co-IP assay using an antibody against Gcm1 showed that an increased binding was found between Gcm1, TCF4, and β-catenin in NTDs brains and spines (Fig. 6f and g). All the results confirmed that increased Gcm1 regulates the Wnt signaling and contributed in neurodevelopment in NTDs mouse models.

Normal and spina bifida fetal brains (20 each) were also selected and analyzed (Table S3). The folate concentration of normal brains was 0.14 ± 0.02 ng/mg, while that of spina bifida was 0.06 ± 0.02 ng/mg (Fig. 6h). Western blot showed that 5.7-folds increase in GCM1 level was observed in low-folate NTDs fetuses brain tissue (Fig. 6i). However, GCM1 was minimally expressed in heart, lungs, and muscle tissue (Fig. S4A), which were consistent with the previously reports. The expression of neural tube closure-related genes, *Axin2*^24^ and *Atoh1*^25,26^were analyzed at protein level by western blotting in 40 brain samples. Surprisingly, the expressions of Axin2 and Atoh1 were 1.4-folds and 1.5-folds higher, respectively, in low-folate NTDs brains (Fig. 6i). Moreover, 12 NTDs samples were used for nanostring experiment, which showed a significant increase in *Gcm1*, *Axin2*, and *Atoh1* transcription level (*P* < 0.001; Fig. 6j). A negative correlation was observed between folate concentration and *Gcm1* mRNA transcription, while the correlations between *Gcm1* mRNA transcription with *Axin2* mRNA expression, and *Atoh1* mRNA expression were both positive (*P* < 0.001, Fig. 6k). Lastly, the Co-IP experiment using an antibody against GCM1 revealed an enhanced interaction between GCM1 and β-catenin in the NTDs brain tissue with low-folate level (Fig. 6l). Taken together, these results demonstrated that abnormal expression of GCM1 modulated Wnt/β-catenin signaling during the development of mammalian nervous system in folate deficiency.

## Discussion

In mammals, Gcm1 is abundant in developing placenta and disappears after the completion of placental morphogenesis^27^. In mice, Gcm1 first appears at approximately E7.5 post conception in the chorionic plate, which forms the inner surface of the developing placenta. Upon completion of the placental structure at E10.5, Gcm1 ceases to be expressed. Targeted mutagenesis of *Gcm1* gene in mice has been shown to cause embryonic lethality because of the failure of placental development approximately 10 days after conception^27^. It suggests that Gcm1 may function in fundamentally different processes for some cell types and is not limited to being a determinant of glial fate. The chicken ortholog of fly gcm1 has been demonstrated to be expressed in early neuronal lineages of the developing chick spinal cord^12^. Ectopic expression of Gcm1 leads to spina bifida in transgenic mice and frequently with a lipoma at the spinal cord, which shared a characteristic with the human spinal dysraphias and suggests human low-axial spina bifida may be related to Gcm1 ectopic expression^28^. However, there was no direct evidence that Gcm1 directly affects neural specification genes to cause neural tube closure failure.

Our study demonstrate that Gcm1 contributes to nervous system development in vertebrates. The data suggested that Gcm1-mediated hyperactivation of Wnt/β-catenin pathway in folate deficiency resembles the signaling of Wnt3a protein in mESCs, as both mechanisms result in inhibition of the expression of anterior neuroectoderm marker Sox1 (Table S2)^29^. As shown in Figure. S2A, cluster analysis according to the microarray-based expression profile demonstrated that NTD patterning and embryonic cranial skeleton morphogenesis were significantly affected by folate deficiency. Previous studies have implied that Wnt signaling pathway is involved in anteroposterior axis specification^30^. In the brain and spine of mouse embryos at E8.5, activation of the Wnt/β-catenin signaling cascade has been visualized, while *Gcm1* mRNA was undetectable. In fact, the canonical Wnt genes are capable of producing NTDs in mice by hyperactivating WNT, while folic acid supplementation can rescue the mice^31^. These findings indicated a direct relationship between folate level and WNT signaling pathway during neurulation. Our study identified robust expression of Gcm1 and Wnt target genes at E9.5 in both the brains and spines of NTD mouse models. This time and these locations were of critical importance for posterior neuropore closure. Furthermore, increased interaction between Gcm1 and β-catenin were found in the brains of NTD fetuses with low-folate levels. It is plausible to believe that overexpression of Gcm1 upregulated Wnt/β-catenin signaling activity during neurulation and resulted in the failure of neural tube closure.

To ascertain why *Gcm1* transcription was upregulated in folate deficiency, the possibility that *Gcm1* may undergo epigenetic regulation mediated by SAM reactions was investigated. However, no different epigenetic regulation in the promoter region of *Gcm1* was found with folate deficiency. Then, we speculated other effectors. Remarkably, Nanog, which binds with *Gcm1* at TBE motifs in the first 1 kb of the *Gcm1* promoter, can positively regulate *Gcm1* transcription under folate deficiency. In low-folate NTDs samples, a significant negative correlation was observed between folate concentration and *Nanog* mRNA expression, while the positive correlation between *Nanog* and *Gcm1* mRNA expressions in low-folate NTDs samples indicated that *Nanog* activated *Gcm1* transcription in low-folate NTDs.

It is noteworthy that we characterized Gcm1 overexpression in the brain of NTD-affected fetuses during 15–36 weeks of gestation. The results indicated an unusual Gcm1 gene transcription, which was maintained over the entire gestational period. Nevertheless. The matching fetal tissues in the first trimeter are less available. This study revealed a previously uncharacterized role of Gcm1 in regulating Wnt signaling pathway in the development of nervous system during mammal embryogenesis. Based on in vivo and in vitro data, we for the first time demonstrated that Gcm1 activated Wnt/β-catenin signaling in low-folate levels, which affects neurodevelopment in mammals. These findings will help to understand how spatiotemporal patterning of Gcm1 regulates and integrates signaling pathways to control cell fate during early mammalian development. In addition, our data will also help to elucidate the mechanism by which folate deficiency causes NTDs during early stages of pregnancy.

## Materials and methods

### Cell culture and folate treatment

C57BL/6 mESCs, KM mouse embryonic fibroblasts (MEFs) and HEK293T cells were kindly provided by the Stem Cell Bank of the Chinese Academy of Sciences (Shanghai, China). NE-4C cells were from ATCC (ATCC number: SCRC-CRL-2925^TM^) and seeded in a plate that had been precoated with poly-L-lysis (Millipore, USA). Folate power (Sigma–Aldrich, USA) was dissolved in Dulbecco’s modified Eagle’s medium (DMEM) 10X without folic acid (Sigma–Aldrich, USA) and with 3.7% filtered sodium hydrogen carbonate (Beijing Chemical Reagent Company, China). The final concentration of 4 mg/L folate as a normal control while the folate-deficient group was treated simply by DMEM without folate. C57BL/6 mESCs were seeded into culture dishes precoated with MEFs cultured in DMEM supplemented with 0.1 mM nonessential amino acids, 0.1 mM glutamate, and 10% fetal bovine serum (FBS) (Invitrogen, USA) overnight. The C57BL/6 mESCs and NE-4C were cultured in the following complete medium: DMEM with or without 4 mg/L folate (Sigma–Aldrich, USA), diluted with UltraPure™ distilled water (Invitrogen, USA), 1 mM glucose, 0.1 mM nonessential amino acids, 0.1 mM glutamate, 15% FBS, 0.1 mM β-mercaptoethanol (Life Technologies, USA), 1000 U/ml leukemia inhibitory factor (Millipore, USA), and 4 mg/L folate. The HEK293T cells were seeded in DMEM with 10% FBS. All cells were maintained at 37 °C in a humidified atmosphere with 5% CO_2_. All cell lines were authenticated by STR profiling and tested as mycoplasma-free.

### Microarray expression profiling

An Agilent Mouse mRNA Array was designed with eight identical arrays per slide (8 × 60 K format) by CapitalBio (Beijing, China), with each array containing probes for approximately 27958 Entrez Gene RNAs and 7419 lncRNAs. The array also contained 1280 Agilent control probes. After six generations of folate deficiency, total RNA was extracted from C57BL/6 mESCs. The RNA was purified, and its concentration was determined based on OD 260/280 readings obtained from a spectrophotometer (NanoDrop ND-1000). RNA integrity was checked by 1% formaldehyde denaturing gel electrophoresis. cDNA labeled with a fluorescent dye (Cy5 or Cy3-dCTP) was produced by Eberwine’s linear RNA amplification method and subsequent enzymatic reaction, in accordance with the manufacturer’s protocol^32^. Three replicates were designed for each experiment. The array data were summarized, normalized, and subjected to quality control using GeneSpring software V13 (Agilent). To define the differentially expressed genes, we used threshold values of an increased expression of ≥2-folds or a decreased to ≤0.5-fold and a *t-*test *P* value of 0.05. The data were log2-transformed and median-centered by gene using the Adjust Data function of Cluster 3.0 software and then further analyzed by hierarchical clustering with average linkage^33^. Finally, we performed tree visualization using Java TreeView (Stanford University School of Medicine, Stanford, CA, USA).

### Bisulfite sequencing

The mESCs samples were treated with bisulfite and the sequences of five CpG islands and three recognition sites in the mouse *Gcm1* gene were amplified. The CpG islands of the genes were determined using online software (http://emboss.bioinformatics.nl/cgi-bin/emboss/cpgplot). Primers were designed using the online software Primer3 (http://frodo.wi.mit.edu/cgi-bin)/primer3/primer3_www.cgi). The PCR products were purified with shrimp enzyme (SAP; Promega, USA) and exonuclease I (EXO I; Epicentre) and sequenced using ABI’s BigDye 3.1 kit (Cat# 4323161, USA). The sequencing reactions were purified with alcohol and loaded into an ABI 3730 instrument.

### Coimmunoprecipitation (Co-IP) assay

The Co-IP assay was performed with a kit from Thermo Fisher (Cat. #26147, USA), following manufacturer’s protocol. For immunoprecipitation, 4 µg of antibody Gcm1(Santa Cruze, USA) or TCF4 (Santa Cruze, USA) was bounded to Protein A/G plus agarose on a rotator at room temperature for 45 min, with IgG used as a negative control. Then crosslinking of bound antibody with 450 µM disuccinimidyl suberate was performed on a rotator at room temperature for 50 min. Simultaneously preclearing of the nuclear protein using control agarose was performed on a rotator at rest at 4 °C for 50 min. The precleared nuclear protein was incubated with the antibody-crosslinked resin overnight at 4 °C to accomplish antigen immunoprecipitation. Antigen elution was then completed on the second day and the bounded proteins were detected by western blotting.

### RNA extraction, reverse transcription, and real-time RT-PCR analysis

Total RNA was extracted from 0.5 × 10^6^ cells using TRIzol reagent in accordance with the manufacturer’s recommendations (Cat. #A33254, TRIzol^TM^ Plus RNA Purification Kit, Invitrogen, USA). Total RNA from various groups were converted into cDNA using TransScript First-Strand cDNA Synthesis SuperMix (TransGen Biotech, China). UltraSYBR Mixture (*CW* Biotech, China) was used for real-time PCR, and quantification of the relative mRNA levels in different cell groups, was then performed using a QuantStudio™ 7 Flex Real-Time PCR system (ABI). Primers were synthesized by CWBiotech, which are listed in Table S4. The reactions mixture contained forward and reverse primers (0.5 µl each), UltraSYBR Mixture (12.5 µl; containing TransStart Taq, SYBR Green I, dNTPs, and PCR enhancer), cDNA (1 µl), and ddH_2_O (10.5 µl). After the initial denaturation step at 95 °C for 3 min, 45 amplification cycles were performed as follows: denaturation at 95 °C for 15 s, annealing at 58 °C for 20 s, and extension at 72 °C for 30 s, followed by a final step of 10 min at 72 °C. The expressions of genes were normalized to that of *Gapdh*. The relative levels of mRNA transcripts were calculated using the classic ΔΔCt method.

### Chromatin immunoprecipitation (ChIP) and ChIP-seq

An Enzymatic Chromatin IP Kit was used for the ChIP assays in accordance with the manufacturer’s protocol (Cat. #9003, SimpleChIP Enzymatic Chromatin IP Kit, Cell Signaling Technology, USA). Formaldehyde-crosslinked chromatin was obtained from ~2 × 10^7^ C57BL/6 mESCs after MEFs had been removed. The crosslinked chromatin was immunoprecipitated with antibodies against Gcm1, TCF4, H3K4me3, H3K27me3, and β-catenin overnight at 4 °C. Nonspecific mouse IgG was used as a negative control.

The immunoprecipitated DNA was analyzed by sequencing. In-depth whole-genome DNA sequencing was performed by CapitalBio Technology (Beijing, China). The raw sequencing data were examined using the illumina analysis pipeline, aligned to the *Mus musculus* reference genome (University of California, Santa Cruz, mm10) using Bowtie2, and further analyzed with the Model-based Analysis of ChIP-Seq (MACS) program (https://github.com/taoliu/MACS). The enriched binding peaks were identified after normalization with the control input.

### Luciferase assay

A TOPFlash construct (400 ng) and a Renilla reporter (400 ng) were separately mixed with a TK control reporter (50 ng) and cotransfected into 10^6^ cells by electroporation. For pGLMV-Gcm1 transfection for Gcm1 overexpression, PGLMV-Gcm1 (4 µg), TOPFlash construct, FOPFlash construct and TK control construct were all mixed together. After 72 h, the Dual-Luciferase® Reporter Assay System (Cat #E2920, Promega, USA) was used to measure the luciferase activity according to the manufacturer’s protocol. The TOP/FOP-flash values were normalized to the Renilla reniformis (Promega, USA) reading and the TOP/FOP ratio was measured.

### Animals

CD-1 mice (7–8 weeks old) were provided by Beijing Vital River laboratory Animal Technology Co., Ltd, China (no: 110011200105514958, 16–25 g). Male and female CD-1 mice were fed on low-folate diet for more than 8 weeks. Sexually matured individuals were mated overnight; vaginal plug was detected at 8:00 am in the following morning and was designated as E0.5 day. NTDs mouse models were induced by intraperitoneal injection with 1.5 mg/kg of MTX (Sigma–Aldrich, USA) on E7.5. On E9.5, pregnant mice were euthanized by cervical dislocation, and the phenotypes of the fetuses were observed under a microscope. All samples with NTDs clinical manifestations were collected for experiments. No specific randomization method was used. All procedures involving animal handling were performed according to the institutional guidelines approved by the Animal Ethics Committee of the Capital Institute of Pediatrics.

### Cell cycle and apoptosis analysis by flow cytometry

For flow cytometry, mESCs were seeded overnight and subjected to folate deficiency for three or six generations. Untreated control cells were simultaneously prepared. C57BL/6 mESCs were harvested, washed, and stained in accordance with the manufacturer’s protocol (Cat. # 340242, BD Cycletest™ Plus DNA Reagent Kit, Becton Dickinson, USA). The cell cycle phase was analyzed using BD CellQuest Pro software with a FACSCalibur™ instrument (Becton Dickinson, USA). For apoptosis analyses, mESCs were trypsinized, washed with PBS, resuspended in PBS, and then supplemented with dropwise ice-cold 100% ethanol to obtain a final ethanol concentration of 75% for fixation overnight at −20 °C. The cells were then centrifuged at 1000 × *g* and 4 °C for 5 min, washed with PBS, and then resuspended in propidium iodide (PI working solution (PBS containing 100 g/ml RNase A, 50 g/ml PI, and 0.1% Triton X-100) for 30 min in the dark at 4 °C. Finally, equal numbers of cells from control and case groups were filtered through a 35 µm strainer cap before being subjected to fluorescence-activated cell sorting (FACS) analysis.

### Small interfering RNAs (siRNAs)

siRNA reagents for Gcm1 and TCF4 were purchased from Santa Cruz Biotechnology (USA). Nanog-siRNA was purchased from Genomeditech (Shanghai, China). Transfection of cells with Gcm1-siRNA and TCF4-siRNA or Nanog-siRNA was performed by electroporation using the Amaxa^TM^Mouse ES Cell NucleofectorTM Kit (Cat. #VPH-1001, Lonza, Sweden). The control group was transfected with scramble siRNA controls in the same way as mentioned above.

### Nanostring

Total RNA was isolated from 24 human brain tissues following manufacturer’s instructions (Cat. #74104, RNeasy Mini Kits, Qiagen, USA). Gene transcripts analysis and specific probes was conducted using the classic MB subtyping gene CodeSet (Nanostring Technologies, USA). Each reaction contained 500 ng of total RNA in a total of 15 ul aliquot, together with hybridization, reporter, and capture probes. The hybridizations were incubated at 65 °C for 18 h, then eluted and immobilized in the cartridge on the nCounter Digital Analyzer for data collection. Raw counts were normalized to internal levels for five reference genes including ACTB, TUBB, CLTC, TBP, and POLR1B. Quality control criteria and analysis of the raw Nanostring data were conducted using nSolver Analysis Software v4.0 (Nanostring Technologies, USA). The Student’s *t*-test was used to compare normalized expression values between normal and NTDs.

### Determination of folate concentrations

The folate concentration of cells and tissues was determined using a competitive receptor binding immunoassay kit (Cat. #A14208, Chemiluminescent Immunoenzyme Assay; Beckman Coulter, USA) and an Access 2 Immunoassay System (Beckman Coulter, USA). Briefly, 10^6^cells or 15 mg of brain tissue were collected in 1 ml of Tris buffer solution, sonicated for nine cycles, and centrifuged at 10,000 rpm for 3 min at 4 °C before the supernatant was tested.

### Subjects

All clinical samples were obtained from the Lvliang city of Shanxi Province in northern China with informed consent from the patients or their families. The enrolled pregnant women were diagnosed by trained clinicians using ultrasonography. The surgical procedures were performed as previously described^34^. The epidemiological method was described in detail in our previous publication^35^. The Ethics Board of Capital Institute of Pediatrics approved the study protocol. Sample size of human NTDs samples was choosen based on G-power calculation. The information of the clinical samples was shown in Table S3.

### Plasmids and antibodies

All plasmids were purchased from Genomeditech (Shanghai, China). pGLMV-GCM1 was subcloned into a pGMLV-6395 expression vector. pNanog-HA was subcloned into a pcDNA3.1 expression vector. The primary antibodies used were: anti-GCM1 (#sc-101173, 1:50; Santa Cruz, USA); anti-β-catenin (#D1018, 1:1000; CST, USA); anti-Nanog (ab214549,1:1000; Abcam, USA); anti-β-catenin (#AF0069, 1:1000; Beyotime, China); anti-TCF4 (sc-166699; 1:500, Santa Cruz, USA); anti-Axin2 (#21515, 1:2000; CST, USA); anti-histone H3 (#ab21054, 1:5000; Abcam, USA); and anti-Atoh1 (#ab168374, 1:1000, Abcam, USA).

### Statistical analysis

All data are presented as the mean ± SEM. Two-tailed Student’s *t*-test was performed to analyze the differences between two groups. Exact sample size(n) is indicated in figure legend of each experiment. All the measurements were repeated at least three times with consistent trends and differences were considered to be statistically significant at *P < 0.05, **P < 0.01, ***P < 0.001. Data point is excluded if it deviates from mean with more than three standard deviations. No variation is estimated in the data of each group. All statistical analyses were performed using SPSS 22.0 software. Variance is similar between the groups that are being statistically compared. Investigators were not blinded to the group allocation during the experiment and when assessing the outcome in all experiments including animal experiments.

## Supplementary information

Supplementary figure legends and table legends

Supplementary Figure 1

Supplementary Figure 2

Supplementary Figure 3

Supplementary Figure 4

Supplementary Table 1

Supplementary Table 2

Supplementary Table 3

Supplementary Table 4

## Data Availability

Expression profile microarray data have been deposited in GEO under accession number GSE124271. The ChIP-seq data have been deposited in GEO under accession number GSE124339.
