# Supplementary material for: Aberrant Gcm1 expression mediates Wnt/β-catenin pathway activation in folate deficiency involved in neural tube defects
Source: Cell Death Dis. 2021 Mar 4;12(3):234. doi: 10.1038/s41419-020-03313-z (PMC7933360; doi:10.1038/s41419-020-03313-z)
Supplement: Supplementary file 1 — Supplementary figure legends and table legends [file 41419_2020_3313_MOESM1_ESM.docx]

**Legends for supplementary materials:**

**Figure supplementary 1: Expression of Gcm1 and genes in Wnt/β-catenin signaling pathway**

(a) Gcm1 expression changes during development from BioGPS. X-axis is different kind of tissue, cell, and different stages of embryo development; y-axis is fragments of kilobase million (FPKM).

(b) β-Catenin protein expression in C57BL/6 mESCs with folate deficiency. Left panel: western blots of β-Catenin; right panel: quantification of β-catenin protein levels.

Data b represents the mean±SEM (n=3). The *p* value was calculated by Student’s *t* test, ns was for no significance, * *P*<0.05, ** *P*<0.01, *** *P*<0.001.

**Figure supplementary 2: Folate deficiency influence embryonic neural-related genes expression**

(a) Heatmap of genes with significant changes in expression upon folate deficiency. Approximately 50 genes with significant differential expression in response to folate deficiency were grouped into four main clusters.

**Figure supplementary 3: Methylation regulation of Gcm1 promoter**

(a) DNA methylation analysis of Gcm1. The arrow represents the site in the CpG of the Gcm1.

(b) ChIP-qPCR analysis of H3K27me3 and H3K4me3 in C57BL/6 mESCs with folate deficiency.

Data represent the mean±SEM (n=3). The *p* value was calculated by Student’s *t* test, ns was for no significance, * *P*<0.05.

**Figure supplementary 4: Gcm1 and Axin2 expression in heart, lungs and muscles of human NTDs samples**

(a) Expression of GCM1 as detected in heart, lungs and muscles of eight randomly selected fetal samples (n=4).

Data represent the mean±SEM (n=4). The *p* value was calculated by Student’s *t* test, ns was for no significance, * *P*<0.05, ** *P*<0.01.

**Supplementary Table 1: NTD Related Genes and Candidate Genes**

**Supplementary Table 2: Expression Change of NTD Related Genes and Candidate Genes in Expression Profile Microarray**

**Supplementary Table 3: Information of Clinical Manifestations of Normal Fetuses and NTD Fetuses and Content of Folate**

**Supplementary Table 4: Primers Used for Real Time RT-PCR and ChIP-qPCR**
