# Supplementary figures and images for: Aberrant Gcm1 expression mediates Wnt/β-catenin pathway activation in folate deficiency involved in neural tube defects
Source: Cell Death Dis. 2021 Mar 4;12(3):234. doi: 10.1038/s41419-020-03313-z (PMC7933360; doi:10.1038/s41419-020-03313-z)

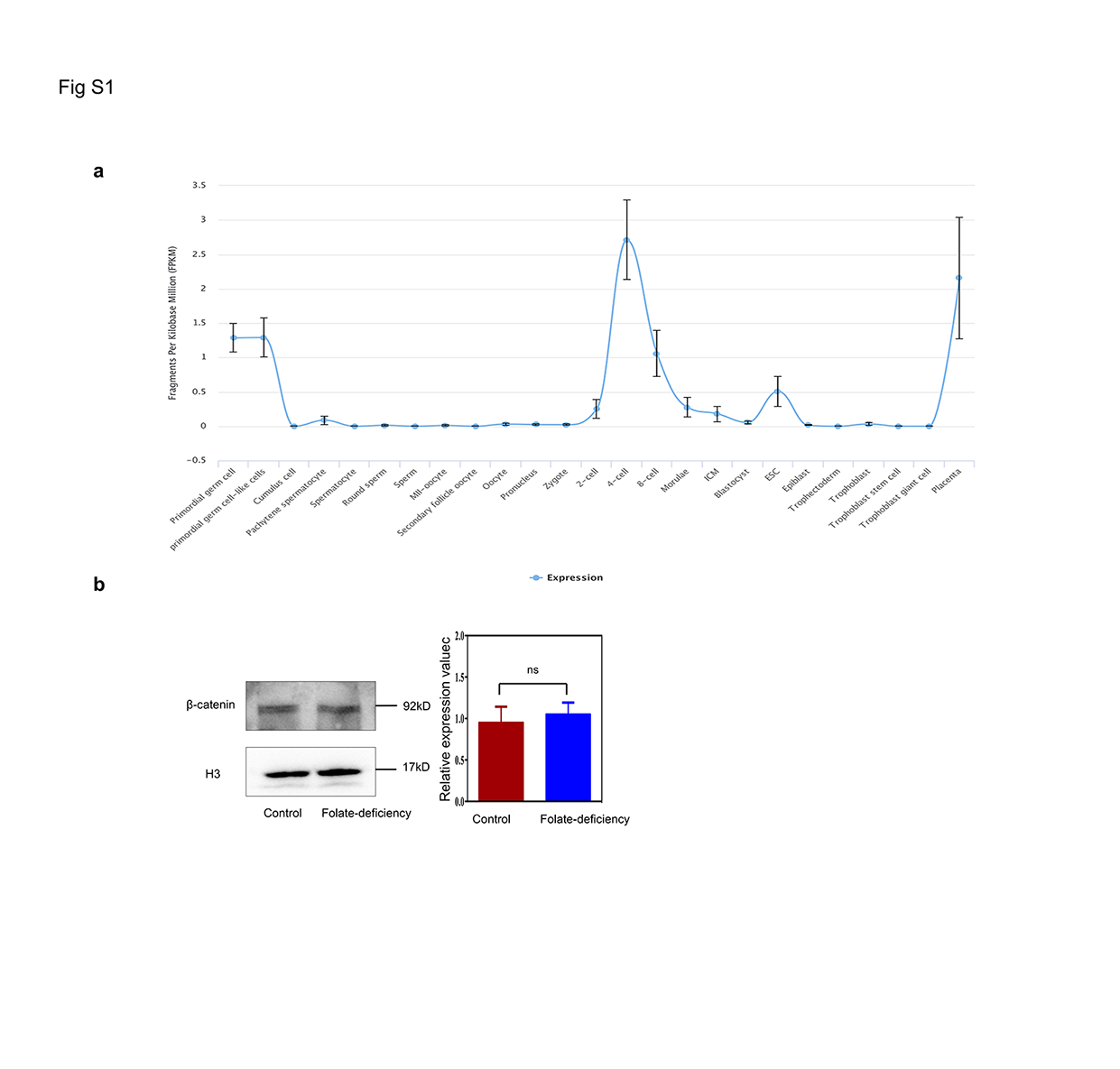

Supplement: Supplementary file 2 — Supplementary Figure 1 [file 41419_2020_3313_MOESM2_ESM.tif]

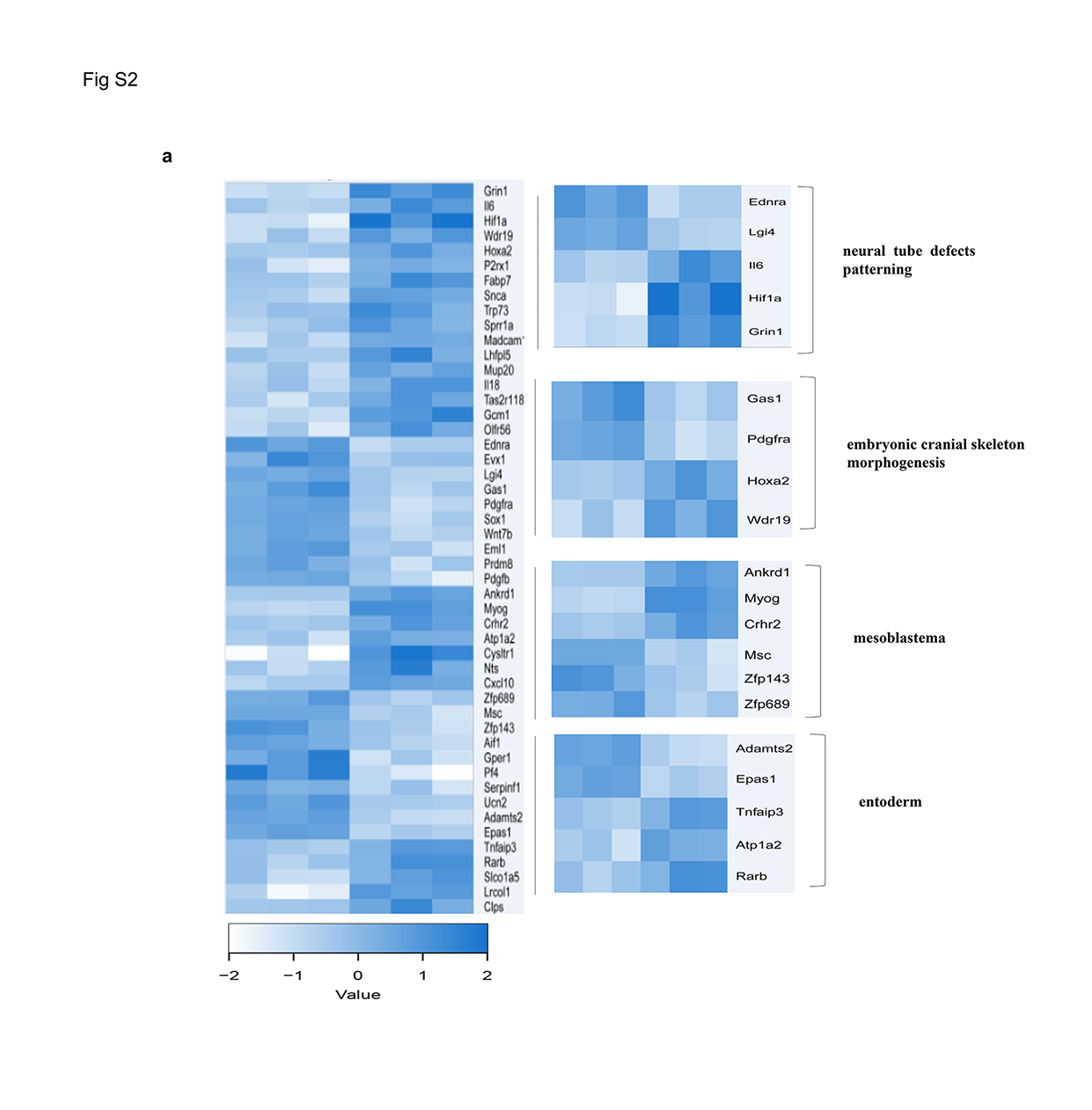

Supplement: Supplementary file 3 — Supplementary Figure 2 [file 41419_2020_3313_MOESM3_ESM.tif]

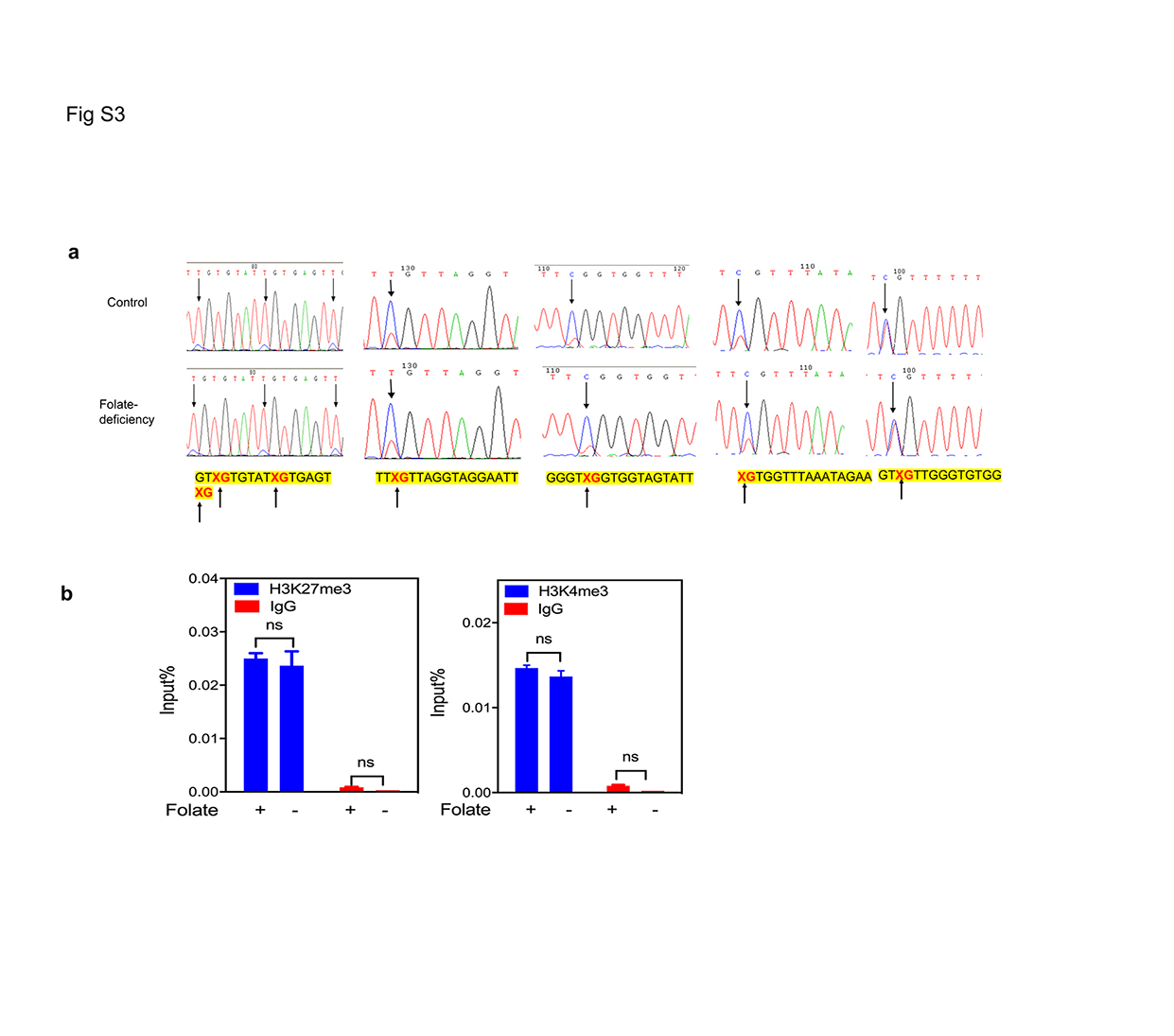

Supplement: Supplementary file 4 — Supplementary Figure 3 [file 41419_2020_3313_MOESM4_ESM.tif]

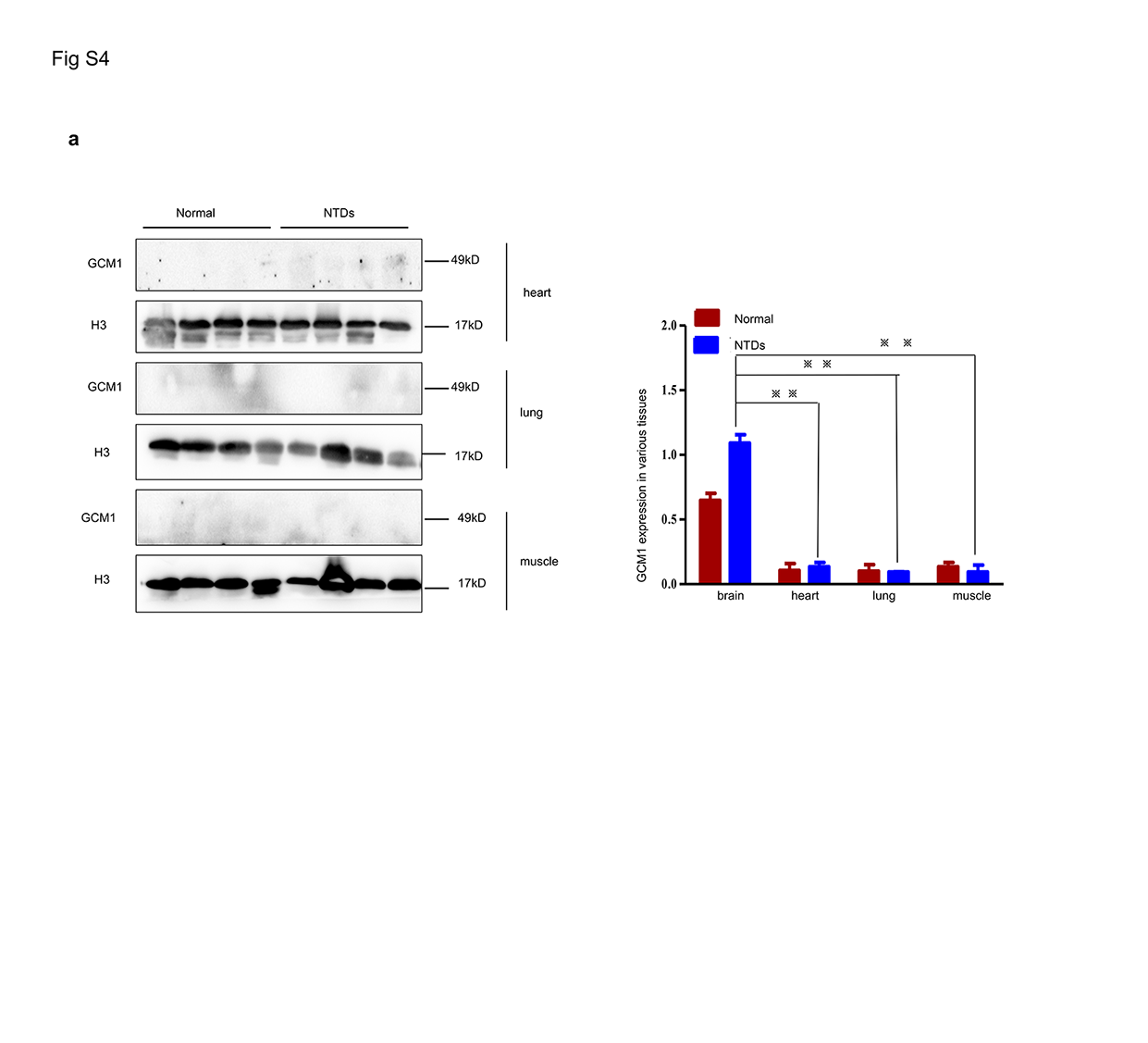

Supplement: Supplementary file 5 — Supplementary Figure 4 [file 41419_2020_3313_MOESM5_ESM.tif]
