# Supplementary material for: Aberrant Gcm1 expression mediates Wnt/β-catenin pathway activation in folate deficiency involved in neural tube defects
Source: Cell Death Dis. 2021 Mar 4;12(3):234. doi: 10.1038/s41419-020-03313-z (PMC7933360; doi:10.1038/s41419-020-03313-z)
Supplement: Supplementary file 6 — Supplementary Table 1 [file 41419_2020_3313_MOESM6_ESM.docx]

Supplementary Table 1: NTD Related Genes and Candidate Genes

| NTDs related genes & candidate genes list | | | | | | | | |
| --- | --- | --- | --- | --- | --- | --- | --- | --- |
| \| MTR \| \| --- \| \| MTHFD1 \| \| FOLH1 \| \| NAT1 \| \| FOLR1 \| \| ALDH1A2 \| \| PAX3 \| \| CITED2 \| \| PDGFRA \| \| TP53 \| \| ZIC3 \| \| SNAI2 \| \| MAPK8 \| \| MAPK9 \| \| VCL \| \| JARID2 \| \| MAP3K4 \| \| EFNA5 \| \| EPHA7 \| \| ALX1 \| \| NAP1L2 \| \| GRHL3 \| \| ATRX \| \| FKBP8 \| \| PTCH1 \| \| RAB23 \| \| SHH \| \| SUFU  INTU  HIRA  FAM4A  MAT2A  SARDH  PCYTIA  PEMT  MGMT \| | \| TULP3 \| \| --- \| \| BMP2 \| \| CTHRC1 \| \| T \| \| VANGL2 \| \| VANGL1 \| \| NUP133 \| \| DNMT3A \| \| DNMT3B \| \| HDAC1 \| \| EP300 \| \| WNT5A \| \| WNT11 \| \| FZD3 \| \| FZD6 \| \| DVL1 \| \| DVL2 \| \| DVL3 \| \| PRICKLE1 \| \| PRICKLE2 \| \| CELSR1 \| \| CELSR2 \| \| CELSR3 \| \| SCRIB \| \| COBL \| \| PTK7 \| \| RHOA \| \| RHO  STIL  SKI  PHACTR4  MAT1A  HK2  MUT  CTH  CHKA \| | \| PAX7 \| \| --- \| \| PAX1 \| \| MAPK8IP1 \| \| TFAP2A \| \| APAF1 \| \| BCL10 \| \| NFKB1 \| \| IKBKB \| \| CHUK \| \| GADD45A \| \| CASP3 \| \| CASP9 \| \| ADNP \| \| CECR2 \| \| GCM1 \| \| HIPK1 \| \| HIPK2 \| \| CREBBP \| \| RYBP \| \| SMARCA4 \| \| SMARCC1 \| \| PAG1 \| \| HECTD1 \| \| MIB2 \| \| SMURF1 \| \| GLI2  KAT2A  SUZ12  STK11  FTCD  NFATC3  GART  BHMT  FLI1  MSX1  MSX2 \| | \| LUZP1 \| \| --- \| \| PRKACB \| \| FGF23 \| \| FGF9 \| \| FGF21 \| \| FGF2 \| \| BMP4 \| \| NOG \| \| BMP1 \| \| PRCP \| \| FRZB \| \| FZD4 \| \| FZD5 \| \| FZD8 \| \| FZD7 \| \| FZD9 \| \| TCN2 \| \| TERC \| \| TFAP2A \| \| UCP2 \| \| CTNNB1 \| \| ITPK1 \| \| PIP5K1C \| \| INPP5E \| \| ABI1 \| \| [ABI2](http://www.ncbi.nlm.nih.gov/gene/10006) \| \| [CRLF1](http://www.ncbi.nlm.nih.gov/gene/9244) \| \| CRLF2 \| \| GRLF1 \| \| MARCKS  SMU  RF2  LEPR  LEP  INSR  INS \| | \| ENAH \| \| --- \| \| VASP \| \| PALLD \| \| SHROOM1 \| \| SHROOM2 \| \| SHROOM3 \| \| SHROOM4 \| \| MARCKSL1 \| \| CSK \| \| BRD2 \| \| HES1 \| \| HES3 \| \| NF1 \| \| NUP50 \| \| NUP188 \| \| NUP85 \| \| NUP155 \| \| POU5F1 \| \| GFAP \| \| SOX1 \| \| DLX2 \| \| NES \| \| NUP98 \| \| NOTCH3 \| \| NUMB \| \| PCMT1 \| \| PRMT1 \| \| PRMT2 \| \| RNMT \| \| NCAM1 \| \| CFL1  MKS1  HK1  SLC2A4  SLC2A1  GAPDH \| | \| APEX1 \| \| --- \| \| BRCA1 \| \| ERCC2 \| \| XRCC1 \| \| XRCC3 \| \| OGG1 \| \| LAMA5 \| \| HSPG2 \| \| ITGAM \| \| ITGA6 \| \| TCOF1 \| \| TXN2 \| \| CRABP1 \| \| CRABP2 \| \| CYP26A1 \| \| RARA \| \| CYP26B1 \| \| MTRR \| \| EZH2 \| \| CBS \| \| TBX1 \| \| ZEB2 \| \| PSEN1 \| \| VEGFA \| \| LRP6 \| \| LHX4 \| \| JAG1 \| \| REST \| \| FGF8 \| \| MIR124 \| \| ATOH1 \| \| GJA1  SLC19A1  FOLR3  TYMS  RFC1 \| | \| MIRN9-1 \| \| --- \| \| ACTL6A \| \| SIX3 \| \| NFATC4 \| \| NTN1 \| \| PPP3R1 \| \| PSEN2 \| \| FOXO1 \| \| GPR161 \| \| IFT172 \| \| TBX20 \| \| TAB2 \| \| TRAF4 \| \| MEF2C \| \| HOXA1 \| \| AFP \| \| HMX3 \| \| LRRC50 \| \| SNRK \| \| SRD5A3 \| \| AMBRA1 \| \| NPAS3 \| \| FOXP4 \| \| CDON \| \| PDGFC \| \| POMT1 \| \| TRPM6 \| \| TSC1 \| \| GDF1 \| \| TWIST1 \| \| BBS4 \| \| CXCR4  FOLR2  MTHFR  DHFR  CUBN \| | \| SP8 \| \| --- \| \| SLC25A19 \| \| GNA13 \| \| ADAM10 \| \| CXCL12 \| \| PTGS2 \| \| PRKCG \| \| PRKCB \| \| GAS1 \| \| COL18A1 \| \| FUZ \| \| CEP290 \| \| IQCB1 \| \| FREM2 \| \| FLRT3 \| \| GMNN \| \| FOXN1 \| \| OFD1 \| \| OPHN1 \| \| FKBP1A \| \| APOB \| \| ZIC1 \| \| ZIC2 \| \| PRKACA \| \| TXN2 \| \| CAT \| \| SOD2 \| \| ALDH1L1 \| \| AMD1 \| \| BHMT2 \| \| AHCY \| \| GAMT  SHMT1  TCN2  TRDMT1 \| |  |
