# Supplementary material for: Aberrant Gcm1 expression mediates Wnt/β-catenin pathway activation in folate deficiency involved in neural tube defects
Source: Cell Death Dis. 2021 Mar 4;12(3):234. doi: 10.1038/s41419-020-03313-z (PMC7933360; doi:10.1038/s41419-020-03313-z)
Supplement: Supplementary file 7 — Supplementary Table 2 [file 41419_2020_3313_MOESM7_ESM.docx]

Supplementary Table 2: Expression Change of NTD Related Genes and Candidate Genes in Expression Profile Microarray

| ProbeName | p (Corr) | p | FC (abs) | Regulation | EntrezGeneID | GeneSymbol |
| --- | --- | --- | --- | --- | --- | --- |
| \| A_51_P130905 \| \| --- \| \| A_51_P345649 \| \| A_55_P2012056 \| \| A_51_P157083 \| \| A_55_P2087013 \| \| A_51_P436201 \| \| A_55_P2055864 \| \| A_55_P2060188 \| \| A_55_P2173927 \| \| A_55_P2036994 \| \| A_51_P411297 \| \| A_52_P249424 \| \| A_55_P1990755 \| \| A_52_P27244 \| \| A_55_P2031736 \| \| A_51_P286748 \| \| A_52_P411601 \| \| A_55_P1962523 \| \| A_51_P106752 \| \| A_51_P510891 \| \| A_51_P380309 \| \| A_65_P20799 \| \| A_52_P509906 \| \| A_52_P157274 \| \| A_55_P2003369 \| \| A_52_P26216 \| \| A_66_P118592 \| \| A_55_P2148873 \| \| A_55_P2041961 \| \| A_55_P2404878 \| \| A_55_P2128646 \| \| A_51_P464576 \| \| A_51_P464738 \| \| A_55_P2055869 \| \| A_55_P2184429 \| \| A_51_P217498 \| \| A_55_P2105181 \| \| A_55_P2029161 \| \| A_55_P2054540 \| \| A_55_P1953545 \| \| A_51_P503131 \| \| A_51_P106059 \| \| A_52_P58145 \| \| A_55_P1966204 \| \| A_55_P2024075 \| \| A_51_P254855 \| \| A_55_P1963580 \| \| A_52_P575178 \| \| A_55_P1973259 \| \| A_55_P2157695 \| \| A_52_P547187 \| \| A_55_P2083654 \| \| A_55_P2078670 \| \| A_51_P296608 \| \| A_65_P20249 \| \| A_55_P2132345 \| \| A_51_P145322 \| \| A_55_P2082403 \| \| A_52_P553316 \| \| A_55_P2060592 \| \| A_55_P2074836 \| \| A_55_P2088425 \| \| A_55_P2032887 \| \| A_52_P18267 \| \| A_55_P2017199 \| \| A_55_P2091861 \| \| A_55_P2186584 \| \| A_55_P1960231 \| \| A_55_P2152962 \| \| A_55_P2006703 \| \| A_55_P1963900 \| \| A_52_P350148 \| \| A_55_P2023391 \| \| A_52_P584335 \| \| A_52_P490272 \| \| A_52_P176160 \| \| A_52_P376360 \| \| A_52_P428496 \| \| A_52_P174915 \| \| A_55_P2031100 \| \| A_55_P2169514 \| \| A_55_P2036999 \| \| A_55_P2083806 \| \| A_55_P2088912 \| \| A_52_P117576 \| \| A_55_P2085425 \| \| A_52_P190973 \| \| A_55_P1979575 \| \| A_55_P1959923 \| \| A_55_P2081133 \| \| A_51_P257550 \| \| A_55_P2051716 \| \| A_52_P603184 \| \| A_55_P1974412 \| \| A_52_P561671 \| \| A_52_P198019 \| \| A_55_P1977653 \| \| A_52_P494514 \| \| A_51_P142107 \| \| A_51_P501844 \| \| A_55_P2101508 \| \| A_51_P348456 \| \| A_66_P133244 \| \| A_55_P1969058 \| \| A_55_P2153461 \| \| A_52_P360515 \| \| A_55_P1973289 \| \| A_55_P2110925 \| \| A_55_P1976282 \| \| A_51_P220162 \| \| A_51_P166456 \| \| A_55_P2028942 \| \| A_55_P2103963 \| \| A_51_P214796 \| \| A_52_P585124 \| \| A_51_P343900 \| \| A_55_P2066463 \| \| A_55_P2124791 \| \| A_51_P416191 \| \| A_55_P1983733 \| \| A_52_P20906 \| \| A_55_P2007228 \| \| A_55_P2015994 \| \| A_66_P130634 \| \| A_55_P1973299 \| \| A_55_P1977929 \| \| A_66_P130100 \| \| A_65_P10399 \| \| A_51_P202340 \| \| A_51_P162671 \| \| A_66_P125962 \| \| A_55_P2145389 \| \| A_52_P342159 \| \| A_51_P481210 \| \| A_55_P2012051 \| \| A_55_P1975903 \| \| A_52_P188690 \| \| A_51_P209527 \| \| A_55_P1959648 \| \| A_55_P2020410 \| \| A_55_P2107667 \| \| A_55_P1954758 \| \| A_66_P121480 \| \| A_55_P2162503 \| \| A_51_P194230 \| \| A_55_P1967295 \| \| A_51_P496381 \| \| A_52_P552665 \| \| A_52_P526372 \| \| A_55_P2032770 \| \| A_55_P1977792 \| \| A_52_P397204 \| \| A_55_P2171086 \| \| A_55_P2165719 \| \| A_55_P2186027 \| \| A_55_P2071858 \| \| A_55_P1958840 \| \| A_55_P2026930 \| \| A_55_P1998154 \| \| A_55_P1970846 \| \| A_55_P2132323 \| \| A_55_P2266295 \| \| A_55_P2027999 \| \| A_55_P2097869 \| \| A_55_P1964812 \| \| A_52_P598605 \| \| A_52_P453785 \| \| A_51_P431737 \| \| A_55_P1972792 \| \| A_55_P2046245 \| \| A_55_P2043987 \| \| A_55_P2041360 \| \| A_66_P117659 \| \| A_55_P2176802 \| \| A_51_P286563 \| \| A_55_P1958246 \| \| A_55_P2154684 \| \| A_51_P480202 \| \| A_51_P365952 \| \| A_51_P363556 \| \| A_55_P1953728 \| \| A_55_P2162935 \| \| A_55_P2118391 \| \| A_55_P2023577 \| \| A_52_P639774 \| \| A_66_P137794 \| \| A_55_P2000548 \| \| A_55_P2037962 \| \| A_55_P2021921 \| \| A_52_P333419 \| \| A_66_P105307 \| \| A_51_P413910 \| \| A_55_P2107750 \| \| A_66_P119643 \| \| A_55_P2087315 \| \| A_55_P2095047 \| \| A_55_P2080956 \| \| A_55_P2107292 \| \| A_55_P2131153 \| \| A_52_P821 \| \| A_52_P449417 \| \| A_55_P1995243 \| \| A_52_P235347 \| \| A_55_P1968738 \| \| A_52_P596008 \| \| A_51_P334739 \| \| A_52_P413947 \| \| A_51_P142923 \| \| A_55_P2116621 \| \| A_55_P2013534 \| \| A_52_P64364 \| \| A_52_P359621 \| \| A_55_P2037425 \| \| A_55_P2033705 \| \| A_55_P2053077 \| \| A_55_P2124619 \| \| A_65_P15689 \| \| A_51_P511015 \| \| A_55_P2130535 \| \| A_52_P312563 \| \| A_55_P1981296 \| \| A_55_P2149983 \| \| A_55_P2041397 \| \| A_55_P2103969 \| \| A_51_P366207 \| \| A_55_P2006698 \| \| A_55_P2091858 \| \| A_55_P1984625 \| \| A_55_P1965836 \| \| A_55_P2118843 \| \| A_55_P2070510 \| \| A_55_P1968743 \| \| A_55_P2151091 \| \| A_55_P1991134 \| \| A_51_P437608 \| \| A_55_P2144556 \| \| A_55_P2075941 \| \| A_51_P483261 \| \| A_55_P2153231 \| \| A_52_P638895 \| \| A_52_P312837 \| \| A_55_P1977934 \| \| A_55_P2101182 \| \| A_55_P2117984 \| \| A_55_P2179587 \| \| A_51_P417701 \| \| A_66_P132888 \| \| A_52_P382149 \| \| A_55_P2003363 \| \| A_51_P396550 \| \| A_55_P2024245 \| \| A_52_P185907 \| \| A_55_P2014978 \| \| A_55_P2153459 \| \| A_52_P329367 \| \| A_55_P2095345 \| \| A_52_P357829 \| \| A_55_P1975793 \| \| A_55_P2037398 \| \| A_52_P544476 \| \| A_55_P2015605 \| \| A_55_P2172566 \| \| A_55_P1997866 \| \| A_55_P1984622 \| \| A_55_P2047639 \| \| A_55_P1976057 \| \| A_51_P472608 \| \| A_52_P588096 \| \| A_55_P2006708 \| \| A_66_P132077 \| \| A_51_P440743 \| \| A_52_P237792 \| \| A_55_P2211341 \| \| A_55_P2052240 \| \| A_55_P2108109 \| \| A_52_P234958 \| \| A_55_P2097548 \| \| A_51_P302167 \| \| A_66_P119191 \| \| A_55_P2043486 \| \| A_55_P2143306 \| \| A_51_P138044 \| \| A_51_P312550 \| \| A_52_P647291 \| \| A_55_P1958245 \| \| A_55_P2015670 \| \| A_55_P2152049 \| \| A_55_P2059352 \| \| A_55_P2023572 \| \| A_51_P413740 \| \| A_55_P2043892 \| \| A_66_P138898 \| \| A_51_P204080 \| \| A_55_P1974587 \| \| A_66_P106418 \| \| A_66_P128199 \| \| A_55_P2018847 \| \| A_55_P1953172 \| \| A_51_P318371 \| \| A_55_P2154387 \| \| A_51_P172502 \| \| A_52_P504787 \| \| A_52_P217710 \| \| A_55_P1970860 \| \| A_55_P2059432 \| \| A_55_P1956223 \| \| A_55_P2009774 \| \| A_55_P2032886 \| \| A_55_P2172560 \| \| A_55_P2124090 \| \| A_52_P55717 \| \| A_55_P2049602 \| \| A_51_P144319 \| \| A_66_P107038 \| \| A_55_P2120577 \| \| A_52_P177373 \| \| A_55_P2178084 \| \| A_55_P2050602 \| \| A_55_P2182324 \| \| A_52_P472660 \| \| A_52_P453884 \| \| A_52_P185343 \| \| A_52_P297009 \| \| A_55_P2157250 \| \| A_55_P2036280 \| \| A_55_P2416119 \| \| A_55_P2000938 \| \| A_55_P1990299 \| \| A_55_P2040748 \| \| A_51_P361220 \| \| A_51_P305547 \| \| A_55_P2040977 \| \| A_55_P2131253 \| \| A_51_P464300 \| \| A_55_P1979929 \| \| A_55_P2118734 \| \| A_55_P2025562 \| \| A_55_P2077608 \| \| A_55_P2035804 \| \| A_51_P500344 \| \| A_55_P2021923 \| \| A_55_P1991505 \| \| A_66_P128384 \| \| A_51_P191611 \| \| A_55_P1990086 \| \| A_51_P288447 \| \| A_51_P237040 \| \| A_55_P2014075 \| \| A_55_P1974788 \| \| A_51_P489903 \| \|  \| \|  \| \|  \| | \| 0.193963422 \| \| --- \| \| 0.249181725 \| \| 0.224583324 \| \| 0.323169203 \| \| 0.572518705 \| \| 0.538671967 \| \| 0.224603856 \| \| 0.922013628 \| \| 0.292124583 \| \| 0.521066417 \| \| 0.813425326 \| \| 0.372719078 \| \| 0.524456355 \| \| 0.526988879 \| \| 0.802144041 \| \| 0.393673211 \| \| 0.786776549 \| \| 0.722730175 \| \| 0.752398963 \| \| 0.441714426 \| \| 0.945809269 \| \| 0.824742276 \| \| 0.893058477 \| \| 0.779490893 \| \| 0.98300112 \| \| 0.684940611 \| \| 0.920786326 \| \| 0.590771067 \| \| 0.488067055 \| \| 0.640243844 \| \| 0.712384697 \| \| 0.752398963 \| \| 0.732231754 \| \| 0.9033182 \| \| 0.938278844 \| \| 0.819645179 \| \| 0.406630537 \| \| 0.490032602 \| \| 0.691352124 \| \| 0.548295909 \| \| 0.934084675 \| \| 0.593168331 \| \| 0.886494413 \| \| 0.572069012 \| \| 0.672932801 \| \| 0.347969592 \| \| 0.667774298 \| \| 0.880320951 \| \| 0.670114401 \| \| 0.802144041 \| \| 0.670114401 \| \| 0.719250611 \| \| 0.69564749 \| \| 0.485032354 \| \| 0.92270314 \| \| 0.815676304 \| \| 0.471967294 \| \| 0.975925832 \| \| 0.80716276 \| \| 0.885707429 \| \| 0.63900078 \| \| 0.644151987 \| \| 0.385676067 \| \| 0.578635861 \| \| 0.537931205 \| \| 0.520241711 \| \| 0.451464075 \| \| 0.34518809 \| \| 0.718727415 \| \| 0.616542157 \| \| 0.646959767 \| \| 0.934926716 \| \| 0.364863619 \| \| 0.964915219 \| \| 0.848093351 \| \| 0.794232554 \| \| 0.426479223 \| \| 0.6505852 \| \| 0.999412851 \| \| 0.706456045 \| \| 0.877950494 \| \| 0.885830798 \| \| 0.597862698 \| \| 0.668951377 \| \| 0.990562808 \| \| 0.376271251 \| \| 0.539406673 \| \| 0.702484473 \| \| 0.512338505 \| \| 0.650607783 \| \| 0.903046387 \| \| 0.681007695 \| \| 0.818544986 \| \| 0.306033681 \| \| 0.943746292 \| \| 0.737971156 \| \| 0.825466371 \| \| 0.878661827 \| \| 0.66528848 \| \| 0.687610345 \| \| 0.770167667 \| \| 0.947019828 \| \| 0.680654483 \| \| 0.884664619 \| \| 0.388779198 \| \| 0.735488098 \| \| 0.656439971 \| \| 0.861395099 \| \| 0.911955803 \| \| 0.843660618 \| \| 0.292934002 \| \| 0.236599413 \| \| 0.381466333 \| \| 0.945809269 \| \| 0.745727779 \| \| 0.566225732 \| \| 0.82045519 \| \| 0.979550265 \| \| 0.673860359 \| \| 0.338297081 \| \| 0.997252977 \| \| 0.454500482 \| \| 0.509430256 \| \| 0.722786001 \| \| 0.736198722 \| \| 0.790454064 \| \| 0.654762345 \| \| 0.991376162 \| \| 0.366466481 \| \| 0.334244437 \| \| 0.616542157 \| \| 0.818544986 \| \| 0.964878689 \| \| 0.415881136 \| \| 0.386343561 \| \| 0.914855497 \| \| 0.393191389 \| \| 0.6684978 \| \| 0.236599413 \| \| 0.955470372 \| \| 0.493690248 \| \| 0.633864874 \| \| 0.470400873 \| \| 0.573971831 \| \| 0.422856688 \| \| 0.800441416 \| \| 0.944432857 \| \| 0.717448566 \| \| 0.72564603 \| \| 0.918498967 \| \| 0.923613865 \| \| 0.317035125 \| \| 0.158716979 \| \| 0.922299802 \| \| 0.757036212 \| \| 0.397017243 \| \| 0.943384786 \| \| 0.274483883 \| \| 0.555240199 \| \| 0.964007145 \| \| 0.987269686 \| \| 0.908537353 \| \| 0.657080395 \| \| 0.894257069 \| \| 0.521066417 \| \| 0.652271198 \| \| 0.490514815 \| \| 0.67109534 \| \| 0.960349463 \| \| 0.745727779 \| \| 0.671180658 \| \| 0.752398963 \| \| 0.938278844 \| \| 0.423326086 \| \| 0.870499011 \| \| 0.554135901 \| \| 0.39156711 \| \| 0.518375645 \| \| 0.999540877 \| \| 0.855820037 \| \| 0.680034249 \| \| 0.893394372 \| \| 0.638640595 \| \| 0.348067145 \| \| 0.998685527 \| \| 0.621079865 \| \| 0.802451761 \| \| 0.293086124 \| \| 0.412100925 \| \| 0.81072202 \| \| 0.794635743 \| \| 0.690341637 \| \| 0.658911059 \| \| 0.573136922 \| \| 0.852289717 \| \| 0.397142635 \| \| 0.405580877 \| \| 0.404105656 \| \| 0.470338601 \| \| 0.532418255 \| \| 0.712359173 \| \| 0.41582794 \| \| 0.567266993 \| \| 0.472877949 \| \| 0.490063517 \| \| 0.88557135 \| \| 0.826834007 \| \| 0.720548312 \| \| 0.978564803 \| \| 0.922013628 \| \| 0.77810924 \| \| 0.389552024 \| \| 0.863385242 \| \| 0.589898202 \| \| 0.593246967 \| \| 0.911955803 \| \| 0.978016046 \| \| 0.944871056 \| \| 0.620647237 \| \| 0.859608616 \| \| 0.765150283 \| \| 0.761430035 \| \| 0.866888845 \| \| 0.827001473 \| \| 0.673860359 \| \| 0.569194984 \| \| 0.267261312 \| \| 0.688571854 \| \| 0.519467616 \| \| 0.572412946 \| \| 0.946584173 \| \| 0.355371397 \| \| 0.992528296 \| \| 0.590771067 \| \| 0.932892877 \| \| 0.532524507 \| \| 0.638533537 \| \| 0.844399068 \| \| 0.728485224 \| \| 0.769603844 \| \| 0.826859802 \| \| 0.82045519 \| \| 0.760155492 \| \| 0.457721986 \| \| 0.367361561 \| \| 0.845812392 \| \| 0.561156771 \| \| 0.640243844 \| \| 0.930821305 \| \| 0.812004211 \| \| 0.788805493 \| \| 0.359281503 \| \| 0.427583359 \| \| 0.369853692 \| \| 0.936016147 \| \| 0.416603937 \| \| 0.393412251 \| \| 0.760844646 \| \| 0.714109348 \| \| 0.942712671 \| \| 0.384923837 \| \| 0.334244437 \| \| 0.597698422 \| \| 0.803481051 \| \| 0.267865515 \| \| 0.747536406 \| \| 0.966310739 \| \| 0.435631337 \| \| 0.671140315 \| \| 0.99647998 \| \| 0.632697735 \| \| 0.70663774 \| \| 0.299061948 \| \| 0.858668583 \| \| 0.438311164 \| \| 0.649193494 \| \| 0.70079437 \| \| 0.557670641 \| \| 0.676862674 \| \| 0.850705986 \| \| 0.437420639 \| \| 0.818332956 \| \| 0.359688386 \| \| 0.85970163 \| \| 0.24836937 \| \| 0.795155426 \| \| 0.294054511 \| \| 0.748794067 \| \| 0.429422254 \| \| 0.811482447 \| \| 0.941407514 \| \| 0.297942223 \| \| 0.767508803 \| \| 0.67020413 \| \| 0.88961968 \| \| 0.324786437 \| \| 0.7579204 \| \| 0.54888418 \| \| 0.794678352 \| \| 0.572980156 \| \| 0.497974574 \| \| 0.539396238 \| \| 0.471269249 \| \| 0.639886884 \| \| 0.78030746 \| \| 0.48141442 \| \| 0.94077234 \| \| 0.676699724 \| \| 0.800638214 \| \| 0.367498947 \| \| 0.425304943 \| \| 0.282075339 \| \| 0.853169623 \| \| 0.412100925 \| \| 0.759294294 \| \| 0.984251956 \| \| 0.656416818 \| \| 0.635239744 \| \| 0.607070073 \| \| 0.784821586 \| \| 0.885313603 \| \| 0.889932324 \| \| 0.567266993 \| \| 0.779316254 \| \| 0.936150783 \| \| 0.978593476 \| \| 0.408624961 \| \| 0.802136579 \| \| 0.48364314 \| \| 0.963490704 \| \| 0.645159933 \| \| 0.713544623 \| \| 0.673525693 \| \| 0.748777601 \| \| 0.703954949 \| \| 0.888733657 \| \| 0.199868302 \| \| 0.833824056 \| \| 0.415881136 \| \| 0.733688303 \| \| 0.459984103 \| \| 0.623909282 \| \| 0.763733131 \| \| 0.723871454 \| \| 0.974459528 \| \| 0.519787981 \| \| 0.572859791 \| \| 0.40339991 \| \| 0.800246397 \| \| 0.662855042 \| \|  \| \|  \| | \| 0.001030669 \| \| --- \| \| 0.00385387 \| \| 0.002175141 \| \| 0.015136437 \| \| 0.198438891 \| \| 0.164242743 \| \| 0.002220552 \| \| 0.790888597 \| \| 0.009038684 \| \| 0.146600532 \| \| 0.564183357 \| \| 0.03248883 \| \| 0.150051027 \| \| 0.153136205 \| \| 0.543017438 \| \| 0.042517065 \| \| 0.513650422 \| \| 0.405511683 \| \| 0.456681199 \| \| 0.078470025 \| \| 0.85381162 \| \| 0.586605433 \| \| 0.730548034 \| \| 0.501120449 \| \| 0.951978194 \| \| 0.347055339 \| \| 0.787601723 \| \| 0.218201996 \| \| 0.117553858 \| \| 0.277429801 \| \| 0.389054155 \| \| 0.456557818 \| \| 0.42205271 \| \| 0.750051679 \| \| 0.834257582 \| \| 0.576550041 \| \| 0.052184508 \| \| 0.118671751 \| \| 0.356201187 \| \| 0.174306343 \| \| 0.823917363 \| \| 0.220588961 \| \| 0.714508057 \| \| 0.197213284 \| \| 0.324348101 \| \| 0.022917126 \| \| 0.314262085 \| \| 0.699893975 \| \| 0.318916622 \| \| 0.542970448 \| \| 0.318920207 \| \| 0.40024642 \| \| 0.362646258 \| \| 0.114678839 \| \| 0.792725403 \| \| 0.569367483 \| \| 0.102316771 \| \| 0.933056284 \| \| 0.552298908 \| \| 0.712671167 \| \| 0.27505657 \| \| 0.281532178 \| \| 0.038159039 \| \| 0.205850058 \| \| 0.163096974 \| \| 0.145192124 \| \| 0.08645671 \| \| 0.021950369 \| \| 0.399803001 \| \| 0.248885926 \| \| 0.285459794 \| \| 0.826511904 \| \| 0.028476535 \| \| 0.905164871 \| \| 0.629729848 \| \| 0.527079155 \| \| 0.068085984 \| \| 0.290380749 \| \| 0.997991168 \| \| 0.380862649 \| \| 0.695425012 \| \| 0.712981186 \| \| 0.225881358 \| \| 0.316641705 \| \| 0.971225247 \| \| 0.033578896 \| \| 0.165244909 \| \| 0.372227472 \| \| 0.138064328 \| \| 0.290536932 \| \| 0.749550367 \| \| 0.340829131 \| \| 0.574982837 \| \| 0.011308071 \| \| 0.846900634 \| \| 0.43162622 \| \| 0.587708239 \| \| 0.696581223 \| \| 0.311058609 \| \| 0.351144915 \| \| 0.486622888 \| \| 0.856771772 \| \| 0.340251907 \| \| 0.710168271 \| \| 0.039539814 \| \| 0.427402289 \| \| 0.297077305 \| \| 0.661167367 \| \| 0.768382991 \| \| 0.62143367 \| \| 0.009335941 \| \| 0.002866692 \| \| 0.036414795 \| \| 0.853972957 \| \| 0.443914331 \| \| 0.190244305 \| \| 0.577776213 \| \| 0.942433246 \| \| 0.32676649 \| \| 0.019182207 \| \| 0.990398606 \| \| 0.089118638 \| \| 0.135467743 \| \| 0.405670206 \| \| 0.429126451 \| \| 0.521175272 \| \| 0.294536551 \| \| 0.974003074 \| \| 0.02924666 \| \| 0.01773609 \| \| 0.24872767 \| \| 0.574953806 \| \| 0.905086878 \| \| 0.060297129 \| \| 0.038330333 \| \| 0.773916802 \| \| 0.041999842 \| \| 0.315870128 \| \| 0.002997893 \| \| 0.879282285 \| \| 0.122126179 \| \| 0.267781051 \| \| 0.101284256 \| \| 0.200191617 \| \| 0.064967066 \| \| 0.538346322 \| \| 0.848887532 \| \| 0.397707959 \| \| 0.411014767 \| \| 0.782792306 \| \| 0.796896503 \| \| 0.013387513 \| \| 0.000364181 \| \| 0.791509708 \| \| 0.464628981 \| \| 0.045092798 \| \| 0.84570284 \| \| 0.005890105 \| \| 0.179864887 \| \| 0.902356203 \| \| 0.963602878 \| \| 0.761058273 \| \| 0.297901198 \| \| 0.732554374 \| \| 0.146566903 \| \| 0.29238104 \| \| 0.118932027 \| \| 0.321085809 \| \| 0.893119188 \| \| 0.443961934 \| \| 0.32152482 \| \| 0.456767147 \| \| 0.834309032 \| \| 0.065292667 \| \| 0.681618758 \| \| 0.179272356 \| \| 0.041474569 \| \| 0.143196565 \| \| 0.998240021 \| \| 0.647356367 \| \| 0.33958834 \| \| 0.731236804 \| \| 0.274379638 \| \| 0.022973378 \| \| 0.995268253 \| \| 0.254514153 \| \| 0.543500772 \| \| 0.009377797 \| \| 0.056335998 \| \| 0.558868182 \| \| 0.528212378 \| \| 0.354960673 \| \| 0.300929137 \| \| 0.199412709 \| \| 0.63918653 \| \| 0.045438666 \| \| 0.051329386 \| \| 0.049615353 \| \| 0.101247259 \| \| 0.158178498 \| \| 0.388591765 \| \| 0.059466823 \| \| 0.192389785 \| \| 0.103134337 \| \| 0.118718777 \| \| 0.712027044 \| \| 0.58973051 \| \| 0.402429722 \| \| 0.940535832 \| \| 0.790963404 \| \| 0.499435915 \| \| 0.040311545 \| \| 0.665301935 \| \| 0.217364726 \| \| 0.220677419 \| \| 0.768195954 \| \| 0.939292705 \| \| 0.850309857 \| \| 0.2537551 \| \| 0.656069897 \| \| 0.478940593 \| \| 0.473652278 \| \| 0.674777461 \| \| 0.590578232 \| \| 0.326707094 \| \| 0.194687425 \| \| 0.004846065 \| \| 0.352596087 \| \| 0.143906994 \| \| 0.197758622 \| \| 0.855847989 \| \| 0.024944351 \| \| 0.976660325 \| \| 0.218213381 \| \| 0.820870909 \| \| 0.158306771 \| \| 0.274301702 \| \| 0.623461176 \| \| 0.416094524 \| \| 0.485664586 \| \| 0.59026378 \| \| 0.577831036 \| \| 0.471477972 \| \| 0.091109391 \| \| 0.029620194 \| \| 0.626449003 \| \| 0.18563691 \| \| 0.27744447 \| \| 0.815436999 \| \| 0.561914778 \| \| 0.518319928 \| \| 0.026717756 \| \| 0.068837042 \| \| 0.031058192 \| \| 0.82903908 \| \| 0.061024797 \| \| 0.042182324 \| \| 0.47285076 \| \| 0.392324045 \| \| 0.843719011 \| \| 0.037736827 \| \| 0.017805214 \| \| 0.22563839 \| \| 0.54536181 \| \| 0.005387466 \| \| 0.447767122 \| \| 0.90832768 \| \| 0.074125889 \| \| 0.321261317 \| \| 0.986803214 \| \| 0.266751799 \| \| 0.381115538 \| \| 0.01041829 \| \| 0.654061174 \| \| 0.07600221 \| \| 0.288327732 \| \| 0.369304435 \| \| 0.182319076 \| \| 0.333444555 \| \| 0.635336715 \| \| 0.075381886 \| \| 0.574291094 \| \| 0.026987022 \| \| 0.656623134 \| \| 0.003498398 \| \| 0.529069888 \| \| 0.009494414 \| \| 0.449661716 \| \| 0.070088729 \| \| 0.560743213 \| \| 0.840623001 \| \| 0.01029561 \| \| 0.48265534 \| \| 0.319256133 \| \| 0.720661947 \| \| 0.015388214 \| \| 0.467431962 \| \| 0.174790101 \| \| 0.528350465 \| \| 0.199078761 \| \| 0.126245525 \| \| 0.16503609 \| \| 0.101785029 \| \| 0.276522458 \| \| 0.502588439 \| \| 0.110672805 \| \| 0.839009645 \| \| 0.333015832 \| \| 0.539700941 \| \| 0.029667591 \| \| 0.066532809 \| \| 0.00733607 \| \| 0.641271623 \| \| 0.056183811 \| \| 0.469681293 \| \| 0.954700566 \| \| 0.29701669 \| \| 0.269945182 \| \| 0.237848211 \| \| 0.509112623 \| \| 0.711627078 \| \| 0.721309289 \| \| 0.191976048 \| \| 0.500894831 \| \| 0.829247124 \| \| 0.940694176 \| \| 0.053924193 \| \| 0.542708686 \| \| 0.113298968 \| \| 0.901118769 \| \| 0.282485034 \| \| 0.391271621 \| \| 0.325578222 \| \| 0.449454165 \| \| 0.375371619 \| \| 0.718770115 \| \| 0.00117638 \| \| 0.603828637 \| \| 0.05948744 \| \| 0.42397073 \| \| 0.092733654 \| \| 0.257683597 \| \| 0.476505793 \| \| 0.407735946 \| \| 0.92814 \| \| 0.144557776 \| \| 0.198880494 \| \| 0.04817842 \| \| 0.537628245 \| \| 0.307375532 \| \|  \| \|  \| \|  \| | \|  \| \| --- \| \| 4.236387269 \| \| 2.637945093 \| \| 2.416207093 \| \| 2.495541942 \| \| 1.181938842 \| \| 1.288152875 \| \| 1.351971305 \| \| 1.044743899 \| \| 1.784405318 \| \| 1.386714611 \| \| 1.07052815 \| \| 1.263934349 \| \| 1.177326639 \| \| 1.4715091 \| \| 1.401516701 \| \| 1.645970542 \| \| 1.509542466 \| \| 1.106240929 \| \| 1.184029241 \| \| 1.751421576 \| \| 1.05203927 \| \| 1.176295261 \| \| 1.159093857 \| \| 1.14805712 \| \| 1.011548291 \| \| 1.28079988 \| \| 1.130322519 \| \| 1.200273277 \| \| 1.321441798 \| \| 1.475182194 \| \| 1.206798008 \| \| 1.08637945 \| \| 1.123823762 \| \| 1.027720533 \| \| 1.098498479 \| \| 1.037226058 \| \| 1.759910548 \| \| 1.796573806 \| \| 1.137445301 \| \| 1.241678708 \| \| 1.038469667 \| \| 1.090999699 \| \| 1.084919431 \| \| 1.488376967 \| \| 1.09177409 \| \| 1.460845941 \| \| 1.331463766 \| \| 1.1017842 \| \| 1.301303759 \| \| 1.100231009 \| \| 1.160951078 \| \| 1.145608372 \| \| 1.177012031 \| \| 1.198853219 \| \| 1.063451947 \| \| 1.19449597 \| \| 1.53186775 \| \| 1.015307309 \| \| 1.139914393 \| \| 1.10255242 \| \| 1.117593218 \| \| 1.158749295 \| \| 1.364582151 \| \| 1.510713439 \| \| 1.350280861 \| \| 1.429153149 \| \| 1.39041424 \| \| 1.224680498 \| \| 1.132229577 \| \| 1.321983136 \| \| 1.105551356 \| \| 1.032662682 \| \| 1.460957043 \| \| 1.023857331 \| \| 1.077111991 \| \| 1.24445116 \| \| 1.22167263 \| \| 1.18203383 \| \| 1.00024 \| \| 1.362079272 \| \| 1.071016063 \| \| 1.062019173 \| \| 1.293605364 \| \| 1.175512676 \| \| 1.010769836 \| \| 1.425683061 \| \| 1.42478955 \| \| 1.211462454 \| \| 1.097804025 \| \| 1.147410929 \| \| 1.029387615 \| \| 1.141877619 \| \| 1.105799185 \| \| 1.281564352 \| \| 1.033012951 \| \| 1.119101474 \| \| 1.071708918 \| \| 1.132742027 \| \| 1.248649624 \| \| 1.192715902 \| \| 1.138328549 \| \| 1.054113855 \| \| 1.408470206 \| \| 1.123929718 \| \| 1.33694818 \| \| 1.186841813 \| \| 1.278957454 \| \| 1.081023059 \| \| 1.133880241 \| \| 1.059467708 \| \| 1.852080389 \| \| 1.552977476 \| \| 1.61265371 \| \| 1.034322511 \| \| 1.162386745 \| \| 1.285558162 \| \| 1.048726645 \| \| 1.018017514 \| \| 1.182261308 \| \| 1.35673326 \| \| 1.001105834 \| \| 1.319253178 \| \| 1.977794707 \| \| 1.21454528 \| \| 1.341270409 \| \| 1.463483506 \| \| 1.290787145 \| \| 1.003098747 \| \| 1.152954871 \| \| 1.391326531 \| \| 1.333256332 \| \| 1.101079614 \| \| 1.019389248 \| \| 1.45645403 \| \| 1.570739844 \| \| 1.070016944 \| \| 1.816720748 \| \| 1.165606831 \| \| 1.957514679 \| \| 1.049443676 \| \| 1.25137698 \| \| 1.098459231 \| \| 1.50492819 \| \| 1.182509463 \| \| 1.734229903 \| \| 1.078981922 \| \| 1.028934914 \| \| 1.101514832 \| \| 1.074264081 \| \| 1.045189189 \| \| 1.035706714 \| \| 1.598013649 \| \| 1.775405834 \| \| 1.028858849 \| \| 1.128660587 \| \| 1.204180053 \| \| 1.038066416 \| \| 1.270403482 \| \| 1.258242076 \| \| 1.035950983 \| \| 1.00880791 \| \| 1.0519648 \| \| 1.179086141 \| \| 1.040070767 \| \| 1.240390993 \| \| 1.076120352 \| \| 1.390215158 \| \| 1.109646715 \| \| 1.024823799 \| \| 1.024025301 \| \| 1.183824616 \| \| 1.210094132 \| \| 1.033081788 \| \| 1.304093737 \| \| 1.060311624 \| \| 1.249671619 \| \| 1.180599195 \| \| 1.223230534 \| \| 1.000528531 \| \| 1.103757607 \| \| 1.265383253 \| \| 1.058299056 \| \| 1.229214429 \| \| 1.359129906 \| \| 1.000847152 \| \| 1.365560716 \| \| 1.103877904 \| \| 1.780217611 \| \| 1.225073267 \| \| 1.256359069 \| \| 1.11036417 \| \| 1.175487609 \| \| 1.223713737 \| \| 1.661875095 \| \| 1.065386257 \| \| 1.769956566 \| \| 1.458685092 \| \| 1.472332706 \| \| 1.336648369 \| \| 2.31397775 \| \| 1.191107786 \| \| 1.172403682 \| \| 1.539347609 \| \| 1.30462309 \| \| 1.831968579 \| \| 1.061297955 \| \| 1.076799985 \| \| 1.178444714 \| \| 1.015105925 \| \| 1.033201675 \| \| 1.129979002 \| \| 1.32245385 \| \| 1.052488695 \| \| 2.549740737 \| \| 1.258650766 \| \| 1.049210921 \| \| 1.022047453 \| \| 1.028001673 \| \| 1.28864145 \| \| 1.097761017 \| \| 1.093399943 \| \| 1.086683409 \| \| 1.042724258 \| \| 1.091329216 \| \| 1.502108123 \| \| 1.505600666 \| \| 1.197597791 \| \| 1.516322794 \| \| 1.158848368 \| \| 1.240532737 \| \| 1.096117214 \| \| 1.694276425 \| \| 1.002277616 \| \| 1.100110607 \| \| 1.019752331 \| \| 1.265093601 \| \| 1.266665659 \| \| 1.066507866 \| \| 1.171480422 \| \| 1.062033406 \| \| 1.122719422 \| \| 1.126123719 \| \| 1.074410137 \| \| 1.18425422 \| \| 1.624154527 \| \| 1.106836666 \| \| 1.362643839 \| \| 1.294594195 \| \| 1.051932699 \| \| 1.097598167 \| \| 1.119102669 \| \| 1.775384952 \| \| 1.180008477 \| \| 1.66444607 \| \| 1.076531387 \| \| 1.205628371 \| \| 1.42138955 \| \| 1.114353146 \| \| 1.199535547 \| \| 1.063083033 \| \| 1.375272783 \| \| 1.456597679 \| \| 1.063096922 \| \| 1.064103734 \| \| 1.683090112 \| \| 1.206057221 \| \| 1.024798466 \| \| 1.399808448 \| \| 1.335847265 \| \| 1.00516762 \| \| 1.311340583 \| \| 1.283950742 \| \| 1.45295458 \| \| 1.062191896 \| \| 1.569132249 \| \| 1.184455427 \| \| 1.152448242 \| \| 1.301158996 \| \| 1.115807561 \| \| 1.145126215 \| \| 1.190751735 \| \| 1.120263906 \| \| 1.342856274 \| \| 1.121994627 \| \| 1.238799437 \| \| 1.072209189 \| \| 1.432243555 \| \| 1.131127266 \| \| 1.451194666 \| \| 1.134497376 \| \| 1.042619637 \| \| 1.796381088 \| \| 1.103369623 \| \| 1.221269817 \| \| 1.08495945 \| \| 1.511807405 \| \| 1.065906081 \| \| 1.404216641 \| \| 1.052765071 \| \| 1.285193391 \| \| 1.636913738 \| \| 1.177150656 \| \| 1.238633248 \| \| 1.188111084 \| \| 1.05825281 \| \| 1.251632397 \| \| 1.026531932 \| \| 1.226804248 \| \| 1.100408544 \| \| 1.394567813 \| \| 1.61822348 \| \| 1.423675252 \| \| 1.078100916 \| \| 1.310447146 \| \| 1.08553067 \| \| 1.015836348 \| \| 1.414856129 \| \| 1.445280599 \| \| 1.316245173 \| \| 1.149625118 \| \| 1.068889871 \| \| 1.054346799 \| \| 1.168912568 \| \| 1.082188846 \| \| 1.041767777 \| \| 1.019676465 \| \| 1.292400207 \| \| 1.195484456 \| \| 1.508762262 \| \| 1.037827525 \| \| 1.137705363 \| \| 1.292407888 \| \| 1.179315374 \| \| 1.083832441 \| \| 1.109014746 \| \| 1.069892518 \| \| 1.343565991 \| \| 1.087359807 \| \| 1.456804709 \| \| 1.110815299 \| \| 1.29876904 \| \| 1.413354943 \| \| 1.056673546 \| \| 1.24379003 \| \| 1.017026396 \| \| 1.27799335 \| \| 1.243975258 \| \| 1.350806111 \| \| 1.130023075 \| \| 2.061894554 \| \| 1.448515 \| \|  \| \|  \| | \|  \| \| --- \| \| up \| \| down \| \| down \| \| down \| \| down \| \| down \| \| down \| \| down \| \| down \| \| down \| \| down \| \| up \| \| down \| \| down \| \| up \| \| down \| \| down \| \| down \| \| down \| \| up \| \| up \| \| down \| \| down \| \| down \| \| up \| \| down \| \| down \| \| down \| \| up \| \| down \| \| up \| \| down \| \| down \| \| up \| \| up \| \| down \| \| up \| \| up \| \| down \| \| down \| \| up \| \| down \| \| up \| \| up \| \| up \| \| up \| \| up \| \| down \| \| down \| \| up \| \| down \| \| down \| \| down \| \| down \| \| up \| \| up \| \| up \| \| up \| \| down \| \| up \| \| down \| \| down \| \| down \| \| down \| \| down \| \| down \| \| down \| \| down \| \| down \| \| down \| \| down \| \| up \| \| down \| \| down \| \| up \| \| down \| \| down \| \| up \| \| down \| \| down \| \| down \| \| up \| \| down \| \| down \| \| down \| \| up \| \| down \| \| up \| \| down \| \| up \| \| down \| \| down \| \| down \| \| down \| \| down \| \| down \| \| down \| \| down \| \| up \| \| down \| \| down \| \| down \| \| down \| \| down \| \| down \| \| down \| \| up \| \| up \| \| down \| \| down \| \| down \| \| down \| \| down \| \| down \| \| down \| \| down \| \| down \| \| up \| \| up \| \| up \| \| down \| \| down \| \| up \| \| down \| \| down \| \| down \| \| down \| \| down \| \| down \| \| up \| \| down \| \| down \| \| up \| \| up \| \| down \| \| down \| \| down \| \| down \| \| down \| \| up \| \| down \| \| down \| \| down \| \| up \| \| up \| \| down \| \| down \| \| down \| \| up \| \| down \| \| up \| \| down \| \| down \| \| down \| \| up \| \| down \| \| down \| \| down \| \| down \| \| down \| \| down \| \| up \| \| down \| \| up \| \| down \| \| down \| \| up \| \| down \| \| up \| \| down \| \| up \| \| down \| \| down \| \| down \| \| down \| \| down \| \| down \| \| up \| \| down \| \| up \| \| up \| \| down \| \| down \| \| down \| \| up \| \| up \| \| down \| \| up \| \| down \| \| down \| \| up \| \| down \| \| down \| \| down \| \| up \| \| down \| \| down \| \| down \| \| down \| \| up \| \| up \| \| down \| \| up \| \| up \| \| up \| \| up \| \| down \| \| down \| \| up \| \| down \| \| down \| \| up \| \| down \| \| up \| \| up \| \| up \| \| up \| \| up \| \| down \| \| down \| \| down \| \| down \| \| up \| \| up \| \| up \| \| down \| \| down \| \| down \| \| down \| \| down \| \| down \| \| down \| \| up \| \| down \| \| down \| \| down \| \| up \| \| up \| \| down \| \| up \| \| down \| \| down \| \| up \| \| down \| \| down \| \| up \| \| up \| \| up \| \| down \| \| up \| \| up \| \| down \| \| down \| \| down \| \| down \| \| down \| \| down \| \| down \| \| down \| \| down \| \| down \| \| down \| \| down \| \| down \| \| up \| \| down \| \| down \| \| down \| \| down \| \| up \| \| down \| \| down \| \| down \| \| up \| \| down \| \| down \| \| down \| \| down \| \| down \| \| up \| \| down \| \| up \| \| down \| \| up \| \| down \| \| down \| \| down \| \| down \| \| down \| \| down \| \| down \| \| up \| \| down \| \| up \| \| up \| \| down \| \| down \| \| up \| \| up \| \| down \| \| up \| \| up \| \| up \| \| down \| \| up \| \| down \| \| up \| \| down \| \| up \| \| down \| \| down \| \| down \| \| down \| \| up \| \| up \| \| up \| \| up \| \| down \| \| down \| \| down \| \| down \| \| up \| \| down \| \| down \| \| down \| \| up \| \| down \| \| down \| \| down \| \| up \| \| up \| \| up \| \| up \| \| down \| \| up \| \| down \| \| down \| \| down \| \| down \| \| down \| \| down \| \| down \| \| up \| \| down \| \| down \| \| down \| \| down \| \| down \| \| down \| \| up \| \| down \| \|  \| \|  \| | \|  \| \| --- \| \| 14531 \| \| 18595 \| \| 20664 \| \| 14451 \| \| 15257 \| \| 14450 \| \| 13543 \| \| 15260 \| \| 16337 \| \| 22773 \| \| 18141 \| \| 22339 \| \| 19058 \| \| 26420 \| \| 27386 \| \| 20378 \| \| 328572 \| \| 22772 \| \| 67897 \| \| 11576 \| \| 17967 \| \| 329165 \| \| 67661 \| \| 11308 \| \| 14232 \| \| 18222 \| \| 22589 \| \| 12359 \| \| 12156 \| \| 227699 \| \| 57441 \| \| 19164 \| \| 20525 \| \| 13543 \| \| 18021 \| \| 20528 \| \| 12116 \| \| 20997 \| \| 12914 \| \| 14312 \| \| 22411 \| \| 22032 \| \| 19378 \| \| 20315 \| \| 18749 \| \| 19225 \| \| 15207 \| \| 57810 \| \| 14431 \| \| 64930 \| \| 68652 \| \| 15468 \| \| 12411 \| \| 13197 \| \| 18751 \| \| 269593 \| \| 18503 \| \| 20473 \| \| 20623 \| \| 15394 \| \| 19099 \| \| 21452 \| \| 18717 \| \| 12189 \| \| 227699 \| \| 15258 \| \| 20425 \| \| 56551 \| \| 238505 \| \| 12153 \| \| 76580 \| \| 53883 \| \| 230824 \| \| 14275 \| \| 269966 \| \| 269378 \| \| 54635 \| \| 11848 \| \| 14609 \| \| 269378 \| \| 64654 \| \| 22773 \| \| 320145 \| \| 14433 \| \| 12367 \| \| 94190 \| \| 22330 \| \| 110380 \| \| 107869 \| \| 14433 \| \| 17357 \| \| 21380 \| \| 24069 \| \| 14534 \| \| 17701 \| \| 70300 \| \| 72333 \| \| 16337 \| \| 14369 \| \| 232174 \| \| 14179 \| \| 19712 \| \| 22330 \| \| 13841 \| \| 330409 \| \| 53883 \| \| 26420 \| \| 71436 \| \| 18752 \| \| 18131 \| \| 14367 \| \| 17967 \| \| 12116 \| \| 52615 \| \| 12767 \| \| 22594 \| \| 13800 \| \| 12822 \| \| 207304 \| \| 107747 \| \| 22160 \| \| 18747 \| \| 14180 \| \| 22171 \| \| 26419 \| \| 16409 \| \| 19335 \| \| 14674 \| \| 18999 \| \| 14276 \| \| 15257 \| \| 71436 \| \| 73181 \| \| 237222 \| \| 20664 \| \| 13436 \| \| 216285 \| \| 12042 \| \| 15530 \| \| 26419 \| \| 13800 \| \| 106042 \| \| 192166 \| \| 57246 \| \| 22771 \| \| 18618 \| \| 71461 \| \| 14369 \| \| 24136 \| \| 64436 \| \| 14275 \| \| 12988 \| \| 72461 \| \| 13026 \| \| 14433 \| \| 17314 \| \| 16403 \| \| 64436 \| \| 19335 \| \| 18618 \| \| 17769 \| \| 14365 \| \| 15275 \| \| 242022 \| \| 21418 \| \| 19206 \| \| 68588 \| \| 107869 \| \| 20460 \| \| 15205 \| \| 269593 \| \| 16974 \| \| 18021 \| \| 15257 \| \| 14674 \| \| 21453 \| \| 20586 \| \| 13392 \| \| 13434 \| \| 329165 \| \| 18008 \| \| 18208 \| \| 16974 \| \| 56551 \| \| 14450 \| \| 240888 \| \| 12631 \| \| 225997 \| \| 14225 \| \| 18021 \| \| 11538 \| \| 15373 \| \| 207304 \| \| 22589 \| \| 71774 \| \| 232087 \| \| 12660 \| \| 19206 \| \| 20588 \| \| 14247 \| \| 229658 \| \| 12808 \| \| 56636 \| \| 18033 \| \| 24136 \| \| 57810 \| \| 17769 \| \| 12660 \| \| 17684 \| \| 67283 \| \| 16150 \| \| 269593 \| \| 14433 \| \| 18505 \| \| 12675 \| \| 66313 \| \| 17850 \| \| 14371 \| \| 13436 \| \| 67897 \| \| 445007 \| \| 74123 \| \| 14056 \| \| 12116 \| \| 17954 \| \| 12153 \| \| 15258 \| \| 20588 \| \| 12914 \| \| 15275 \| \| 14451 \| \| 18033 \| \| 100169 \| \| 14225 \| \| 22158 \| \| 71436 \| \| 18015 \| \| 99011 \| \| 20481 \| \| 22339 \| \| 20588 \| \| 16409 \| \| 18537 \| \| 93840 \| \| 107934 \| \| 11783 \| \| 13361 \| \| 13082 \| \| 14232 \| \| 24069 \| \| 17769 \| \| 12903 \| \| 13544 \| \| 330409 \| \| 12660 \| \| 19401 \| \| 14633 \| \| 57357 \| \| 15275 \| \| 21748 \| \| 74123 \| \| 16337 \| \| 238505 \| \| 20588 \| \| 65969 \| \| 66313 \| \| 93840 \| \| 380614 \| \| 12153 \| \| 320299 \| \| 12614 \| \| 20509 \| \| 26407 \| \| 12931 \| \| 14179 \| \| 13542 \| \| 14433 \| \| 12808 \| \| 64436 \| \| 17702 \| \| 19712 \| \| 56458 \| \| 227699 \| \| 330409 \| \| 21453 \| \| 16403 \| \| 74335 \| \| 12822 \| \| 56551 \| \| 14317 \| \| 57357 \| \| 320145 \| \| 15277 \| \| 229658 \| \| 18749 \| \| 14367 \| \| 57914 \| \| 15218 \| \| 210009 \| \| 12159 \| \| 20315 \| \| 13841 \| \| 14368 \| \| 18618 \| \| 12904 \| \| 12660 \| \| 22771 \| \| 18717 \| \| 57441 \| \| 228361 \| \| 11538 \| \| 14534 \| \| 13640 \| \| 72461 \| \| 17684 \| \| 380614 \| \| 13435 \| \| 17967 \| \| 13800 \| \| 66313 \| \| 56458 \| \| 14674 \| \| 217837 \| \| 14580 \| \| 19165 \| \| 216274 \| \| 269378 \| \| 94190 \| \| 105782 \| \| 14366 \| \| 20583 \| \| 27386 \| \| 110380 \| \| 14559 \| \| 72461 \| \| 67897 \| \| 14451 \| \| 433759 \| \| 14370 \| \| 232087 \| \| 14225 \| \| 14431 \| \| 433759 \| \| 12359 \| \| 380718 \| \| 234865 \| \| 18121 \| \| 13871 \| \| 12631 \| \| 18294 \| \| 71950 \| \|  \| \|  \| | \|  \| \| --- \| \| Gcm1 \| \| Pdgfra \| \| Sox1 \| \| Gas1 \| \| Hipk1 \| \| Gart \| \| Dvl2 \| \| Hira \| \| Insr \| \| Zic3 \| \| Nup50 \| \| Vegfa \| \| Ppp3r1 \| \| Mapk9 \| \| Npas3 \| \| Frzb \| \| Ep300 \| \| Zic2 \| \| Rnmt \| \| Afp \| \| Ncam1 \| \| Abi2 \| \| Ift172 \| \| Abi1 \| \| Fkbp8 \| \| Numb \| \| Atrx \| \| Cat \| \| Bmp2 \| \| Nup188 \| \| Gmnn \| \| Psen1 \| \| Slc2a1 \| \| Dvl2 \| \| Nfatc3 \| \| Slc2a4 \| \| Bhmt \| \| T \| \| Crebbp \| \| Brd2 \| \| Wnt11 \| \| Traf4 \| \| Aldh1a2 \| \| Cxcl12 \| \| Prkacb \| \| Ptgs2 \| \| Hes3 \| \| Cdon \| \| Gamt \| \| Tsc1 \| \| Tab2 \| \| Prmt2 \| \| Cbs \| \| Gadd45a \| \| Prkcb \| \| Luzp1 \| \| Pax1 \| \| Six3 \| \| Snrk \| \| Hoxa1 \| \| Mapk8ip1 \| \| Tcn2 \| \| Pip5k1c \| \| Brca1 \| \| Nup188 \| \| Hipk2 \| \| Shmt1 \| \| Txn2 \| \| Mtr \| \| Bmp1 \| \| Mib2 \| \| Celsr2 \| \| Grhl3 \| \| Folr1 \| \| Nup98 \| \| Ahcy \| \| Pdgfc \| \| Rhoa \| \| Gja1 \| \| Ahcy \| \| Fgf23 \| \| Zic3 \| \| Sp8 \| \| Gapdh \| \| Casp3 \| \| Ophn1 \| \| Vcl \| \| Shroom2 \| \| Cth \| \| Gapdh \| \| Marcksl1 \| \| Tbx1 \| \| Sufu \| \| Kat2a \| \| Msx1 \| \| Fuz \| \| Palld \| \| Insr \| \| Fzd7 \| \| Cyp26b1 \| \| Fgf8 \| \| Rest \| \| Vcl \| \| Epha7 \| \| Cecr2 \| \| Celsr2 \| \| Mapk9 \| \| Flrt3 \| \| Prkcg \| \| Notch3 \| \| Fzd5 \| \| Ncam1 \| \| Bhmt \| \| Suz12 \| \| Cxcr4 \| \| Xrcc1 \| \| Enah \| \| Col18a1 \| \| Hectd1 \| \| Aldh1l1 \| \| Twist1 \| \| Prkaca \| \| Fgf9 \| \| Tyms \| \| Mapk8 \| \| Itgam \| \| Rab23 \| \| Gna13 \| \| Pou5f1 \| \| Folr2 \| \| Hipk1 \| \| Flrt3 \| \| Nfatc4 \| \| Ofd1 \| \| Sox1 \| \| Dnmt3b \| \| Alx1 \| \| Bcl10 \| \| Hspg2 \| \| Mapk8 \| \| Enah \| \| Prickle1 \| \| Sardh \| \| Tbx20 \| \| Zic1 \| \| Pemt \| \| Ptk7 \| \| Fzd7 \| \| Zeb2 \| \| Inpp5e \| \| Folr1 \| \| Csk \| \| Prcp \| \| Pcyt1a \| \| Gapdh \| \| Mgmt \| \| Itga6 \| \| Inpp5e \| \| Rab23 \| \| Pemt \| \| Mthfr \| \| Fzd3 \| \| Hk1 \| \| Frem2 \| \| Tfap2a \| \| Ptch1 \| \| Cthrc1 \| \| Cth \| \| Stil \| \| Hes1 \| \| Luzp1 \| \| Lrp6 \| \| Nfatc3 \| \| Hipk1 \| \| Gna13 \| \| Tcof1 \| \| Smarca4 \| \| Dlx2 \| \| Trdmt1 \| \| Abi2 \| \| Nes \| \| Ntn1 \| \| Lrp6 \| \| Txn2 \| \| Gart \| \| Gpr161 \| \| Cfl1 \| \| Trpm6 \| \| Fkbp1a \| \| Nfatc3 \| \| Adnp \| \| Hmx3 \| \| Hectd1 \| \| Atrx \| \| Shroom1 \| \| Mat2a \| \| Chka \| \| Ptch1 \| \| Smarcc1 \| \| Fli1 \| \| Vangl1 \| \| Cobl \| \| Fgf21 \| \| Nfkb1 \| \| Zeb2 \| \| Cdon \| \| Mthfr \| \| Chka \| \| Cited2 \| \| Slc25a19 \| \| Ikbkb \| \| Luzp1 \| \| Gapdh \| \| Pax3 \| \| Chuk \| \| Smurf2 \| \| Mut \| \| Fzd9 \| \| Dnmt3b \| \| Rnmt \| \| Nup85 \| \| Foxp4 \| \| Ezh2 \| \| Bhmt \| \| Nap1l2 \| \| Bmp1 \| \| Hipk2 \| \| Smarcc1 \| \| Crebbp \| \| Hk1 \| \| Gas1 \| \| Nfkb1 \| \| Phactr4 \| \| Fkbp1a \| \| Tulp3 \| \| Flrt3 \| \| Nf1 \| \| Pomt1 \| \| Ski \| \| Vegfa \| \| Smarcc1 \| \| Itgam \| \| Pcmt1 \| \| Vangl2 \| \| Celsr3 \| \| Apaf1 \| \| Dhfr \| \| Cyp26a1 \| \| Fkbp8 \| \| Sufu \| \| Mthfr \| \| Crabp1 \| \| Dvl3 \| \| Cecr2 \| \| Chka \| \| Rara \| \| Gli2 \| \| Srd5a3 \| \| Hk1 \| \| Terc \| \| Foxp4 \| \| Insr \| \| Mtr \| \| Smarcc1 \| \| Cubn \| \| Smurf2 \| \| Vangl2 \| \| Intu \| \| Bmp1 \| \| Iqcb1 \| \| Celsr1 \| \| Slc19a1 \| \| Map3k4 \| \| Crlf1 \| \| Fgf8 \| \| Dvl1 \| \| Gapdh \| \| Cobl \| \| Inpp5e \| \| Msx2 \| \| Rest \| \| Foxo1 \| \| Nup188 \| \| Cecr2 \| \| Tcof1 \| \| Itga6 \| \| Xrcc3 \| \| Col18a1 \| \| Txn2 \| \| Ftcd \| \| Srd5a3 \| \| Sp8 \| \| Hk2 \| \| Vangl1 \| \| Prkacb \| \| Fzd5 \| \| Crlf2 \| \| Foxn1 \| \| Mtrr \| \| Bmp4 \| \| Cxcl12 \| \| Epha7 \| \| Fzd6 \| \| Pemt \| \| Crabp2 \| \| Chka \| \| Zic1 \| \| Pip5k1c \| \| Gmnn \| \| Ambra1 \| \| Adnp \| \| Kat2a \| \| Efna5 \| \| Prcp \| \| Cited2 \| \| Intu \| \| Dnmt3a \| \| Ncam1 \| \| Enah \| \| Smurf2 \| \| Foxo1 \| \| Gna13 \| \| Itpk1 \| \| Gfap \| \| Psen2 \| \| Cep290 \| \| Ahcy \| \| Ophn1 \| \| Scrib \| \| Fzd4 \| \| Snai2 \| \| Npas3 \| \| Shroom2 \| \| Gdf1 \| \| Prcp \| \| Rnmt \| \| Gas1 \| \| Hdac1 \| \| Fzd8 \| \| Mat2a \| \| Fkbp1a \| \| Gamt \| \| Hdac1 \| \| Cat \| \| Mks1 \| \| Nup133 \| \| Nog \| \| Ercc2 \| \| Cfl1 \| \| Ogg1 \| \| Nanog \| \|  \| \|  \| |
