# Supplementary material for: Aberrant Gcm1 expression mediates Wnt/β-catenin pathway activation in folate deficiency involved in neural tube defects
Source: Cell Death Dis. 2021 Mar 4;12(3):234. doi: 10.1038/s41419-020-03313-z (PMC7933360; doi:10.1038/s41419-020-03313-z)
Supplement: Supplementary file 8 — Supplementary Table 3 [file 41419_2020_3313_MOESM8_ESM.docx]

Table S3 Clinical manifestations of normal fetuses and NTD fetuses

| Sample type | tissue | Folate of brain(ng/mg) | Gender | Gestational  Weeks |
| --- | --- | --- | --- | --- |
| Normal | Brain | 0.39 | female | 22 |
| Normal | Brain | 0.153 | female | 36 |
| Normal | Brain | 0.24 | female | 26 |
| Normal | Brain | 0.03 | male | 21 |
| Normal | Brain | 0.022 | male | 25 |
| Normal | Brain | 0.1 | female | 22 |
| Normal | Brain | 0.2 | male | 26 |
| Normal | Brain | 0.081 | female | 25 |
| Normal | Brain | 0.12 | male | 18 |
| Normal | Brain | 0.110 | female | 15 |
| Normal | Brain | 0.089 | male | 23 |
| Normal | Brain | 0.108 | male | 16 |
| Normal | Brain | 0.041 | female | 24 |
| Normal | Brain | 0.213 | female | 22 |
| Normal | Brain | 0.088 | female | 23 |
| Normal | Brain | 0.46 | male | 24 |
| Normal | Brain | 0.109 | female | 24 |
| Normal | Brain | 0.110 | male | 27 |
| Normal | Brain | 0.098 | male | 24 |
| Normal | Brain | 0.168 | female | 26 |
| Spina bifida | Brain | 0.25 | female | 20 |
| Spina bifida | Brain | 0.013 | female | 33 |
| Spina bifida | Brain | 0.059 | female | 27 |
| Spina bifida | Brain | 0.011 | male | 22 |
| Spina bifida | Brain | 0.062 | male | 16 |
| Spina bifida | Brain | 0.083 | female | 21 |
| Spina bifida | Brain | 0.051 | male | 19 |
| Spina bifida | Brain | 0.024 | female | 23 |
| Spina bifida | Brain | 0.064 | female | 24 |
| Spina bifida | Brain | 0.068 | female | 19 |
| Spina bifida | Brain | 0.035 | male | 16 |
| Spina bifida | Brain | 0.058 | male | 21 |
| Spina bifida | Brain | 0.075 | female | 15 |
| Spina bifida | Brain | 0.055 | male | 18 |
| Spina bifida | Brain | 0.11 | female | 20 |
| Spina bifida | Brain | 0.0375 | female | 26 |
| Spina bifida | Brain | 0.12 | female | 24 |
| Spina bifida | Brain | 0.1 | male | 22 |
| Spina bifida | Brain | 0.065 | male | 20 |
| Spina bifida | Brain | 0.042 | female | 21 |
