# Supplementary material for: Aberrant Gcm1 expression mediates Wnt/β-catenin pathway activation in folate deficiency involved in neural tube defects
Source: Cell Death Dis. 2021 Mar 4;12(3):234. doi: 10.1038/s41419-020-03313-z (PMC7933360; doi:10.1038/s41419-020-03313-z)
Supplement: Supplementary file 9 — Supplementary Table 4 [file 41419_2020_3313_MOESM9_ESM.docx]

Supplementary Table S4: Primers used for Real Time RT-PCR and ChIP-qPCR

| Name | Forward/  Reverse | Sequence(5'to3') | Category |
| --- | --- | --- | --- |
| Gcm1 | Forward | TGTCGTCCGAGCTGTAGATG | RT-PCR |
|  | Reverse | AACGATGTGAAACTGCCTCA | RT-PCR |
| Axin2 | Forward | TGCATCTCTCTCTGGAGCTG | RT-PCR |
|  | Reverse | ACAGCGAGTTATCCAGCGAC | RT-PCR |
| Lef1 | Forward | CTCGTCGCTGTAGGTGATGA | RT-PCR |
|  | Reverse | AAATGGGTCCCTTTCTCCAC | RT-PCR |
| Bcl9l | Forward | CGAAGGGTGGGTATCTGTGT | RT-PCR |
|  | Reverse | TACTGAAGGGCCAGGTTGAA | RT-PCR |
| Tcf4 | Forward | TCTCCATAGTTCCTGGACGG | RT-PCR |
|  | Reverse | GTGGACATTTCACTGGCTCA | RT-PCR |
| Tcf12 | Forward | TCCCCAAGATGTTGTTCCTC | RT-PCR |
|  | Reverse | GTGCGATGTTTTCTCCACCT | RT-PCR |
| Fzd5 | Forward | CTCCGACTCCAAGGACAGAA | RT-PCR |
|  | Reverse | TTGTCGTTAAACTTTCCCAGC | RT-PCR |
| Lrp5 | Forward | ACTCCAGCTTCACTCCGC | RT-PCR |
|  | Reverse | CTGTACTGCAGCTTGGTCCC | RT-PCR |
| Wnt6 | Forward | CCTGCAGATGCTGGTAGGAT | RT-PCR |
|  | Reverse | ACTGCTGCTGCTGCTCTTGT | RT-PCR |
| Ccnd1 | Forward | GGGTGGGTTGGAAATGAAC | RT-PCR |
|  | Reverse | TCCTCTCCAAAATGCCAGAG | RT-PCR |
| Nfat5 | Forward | GAGGGGTGTGGATTGGAATCT | RT-PCR |
|  | Reverse | CTGGTGCTCATGTTACTGAAGTT | RT-PCR |
| Clta4 | Forward | CATGGTGTCGCCAGCTTTC | RT-PCR |
|  | Reverse | GGTAATCTAGGAAGCCCACTGTA | RT-PCR |
| Edn1 | Forward | TTTCCCGTGATCTTCTCTCTGC | RT-PCR |
|  | Reverse | CTGAGTTCGGCTCCCAAGAC | RT-PCR |
| Gapdh | Forward | AATGGATTTGGACGCATTGGT | RT-PCR |
|  | Reverse | TTTGCACTGGTACGTGTTGAT | RT-PCR |
| Nanog | Forward | CACAGTTTGCCTAGTTCTGAGG | RT-PCR |
|  | Reverse | GCAAGAATAGTTCTCGGGATGAA | RT-PCR |
|  | Reverse | ACACACACACACACACACTC | ChIP-qPCR |
| Gcm1 | Forward | AGAAAATCCGATACCCGGAG | ChIP-qPCR |
|  | Reverse | AGAGGAGGAGCTTGAGGGAG | ChIP-qPCR |
| Axin2-WRE | Forward | CGTATGTGTGGGAGGCATT | ChIP-qPCR |
|  | Reverse | GCACACTCATGGACCTCTTT | ChIP-qPCR |
| Axin2-NEG | Forward | TGGTTTTCCCAAATCTTCGCA | ChIP-qPCR |
|  | Reverse | CAGGGCTATCTCTTCAACCCC | ChIP-qPCR |
| Bcl9l-WRE1 | Forward | TGAGGTGTCTGTGTTTGTGTAA | ChIP-qPCR |
|  | Reverse | ACACAAGGGAGCCATAGAAAG | ChIP-qPCR |
| Bcl9l-WRE2 | Forward | GTCAGCTTGTTGGTGTCTCT | ChIP-qPCR |
|  | Reverse | GATAGCCGGTGGACTCATTG | ChIP-qPCR |
| Bcl9l-NEG | Forward | ATGTGGGTGTTGGGAAGCAA | ChIP-qPCR |
|  | Reverse | ATTGGAAAGTTGGCTCAGCG | ChIP-qPCR |
| Isl1-WRE1 | Forward | GGAACCAACCTCGGAGTTAAT | ChIP-qPCR |
|  | Reverse | ATCTGAGCTCTCCAAAGACTTG | ChIP-qPCR |
| Isl1-WRE2 | Forward | CGTTTGACCTTTGCGTTTCC | ChIP-qPCR |
|  | Reverse | CCGTGTTGACCCTGTTACTC | ChIP-qPCR |
|  | Reverse | CTCTGGCAACTGTCCAATCA | ChIP-qPCR |
| Isl1-NEG | Forward | TTCCTTCAATTCCCACCCAGG | ChIP-qPCR |
|  | Reverse | GAAGGCAACCTGCGATTTGTT | ChIP-qPCR |
| Clta4 | Forward | CATGGTGTCGCCAGCTTTC | ChIP-qPCR |
|  | Reverse | GGTAATCTAGGAAGCCCACTGTA | ChIP-qPCR |
| Edn1 | Forward | TTTCCCGTGATCTTCTCTCTGC | ChIP-qPCR |
|  | Reverse | TGAGTTCGGCTCCCAAGAC | ChIP-qPCR |
